# Machine learning to predict osteoradionecrosis in head and neck cancer

**DOI:** 10.3389/fonc.2026.1955800

**Published:** 2026-10-21

**Authors:** Vitoria Tavares Castro, Luan Matheus Trindade Dalmazo, Paula Elaine Diniz Dos Reis, Wenzel Castro de Abreu, Ricardo Gomes dos Reis, Ana Carolina Prado-Ribeiro, André Ferreira Leite, Fabiana T. Almeida, Eliete Neves Silva Guerra

**Affiliations:** 1 University of Brasilia, Laboratory of Oral Histopathology, School of Health Sciences, Brasília, Brazil; 2 Department of Informatics, Federal University of Parana, Curitiba, Paraná, Brazil; 3 University of Brasilia, Interdisciplinary Laboratory of Research Applied to Clinical Practice in Oncology, Nursing Department, School of Health Sciences, Brasília, Brazil; 4 High-Complexity Oncology Care Center, University Hospital of Brasilia, Brasília, Brazil; 5 Dental Oncology Service, Instituto Do Câncer Do Estado de São Paulo, Faculdade de Medicina da Universidade de São Paulo, São Paulo, Brazil; 6 Bioengineering Graduate Program, Scientific and Technological Institute, Brazil University, São Paulo, Brazil; 7 Mike Petryk School of Dentistry, Faculty of Medicine and Dentistry, University of Alberta, Edmonton, AB, Canada

**Keywords:** head and neck neoplasms, machine learning, osteoradionecrosis, predictive learning models, radiotherapy

## Abstract

**Objective:**

Osteoradionecrosis is one of the most severe late adverse effects of head and neck radiotherapy. Predictive models may help identify patients at increased risk and support personalized treatment. This exploratory study aimed to develop a machine learning–based prediction model for osteoradionecrosis in a real-world cohort, following the TRIPOD+AI statement.

**Methods:**

Clinical variables and mandibular dose–volume histogram parameters were collected from head and neck cancer patients treated with radiotherapy. Forward Stepwise Logistic Regression was used for predictor selection. Six machine learning algorithms, Logistic Regression, Random Forest, Support Vector Machine, CatBoost, XGBoost, and TabPFN, were evaluated under class-weighted baseline modelling, threshold optimization, and data augmentation with repeated stratified 5-fold cross-validation. Performance was primarily assessed using sensitivity. Pairwise comparisons used the Nadeau–Bengio corrected paired t-test with Holm adjustment.

**Results:**

Ninety-seven patients were included, of whom 8 developed osteoradionecrosis (8.25%). Of the 80 available variables, V60Gy and tumor location in the oral cavity were selected as predictors. Without augmentation, Logistic Regression achieved the highest sensitivity (0.74 [95% CI: 0.40–1.00]), while several algorithms showed poor sensitivity. With data augmentation, sensitivity ranged from 0.72 to 0.77 across models, with TabPFN, CatBoost, Logistic Regression and Support Vector Machine reaching the highest sensitivity. Logistic Regression achieved the highest AUC under both strategies (0.87). However, F1 scores remained modest, reaching a maximum of 0.48.

**Conclusion:**

Machine learning models demonstrated exploratory potential for ORN risk assessment within an internal validation framework. Class-imbalance strategies were associated with greater minority-class detection in this cohort, but their generalizability remains uncertain. Larger multicenter cohorts and external validation are needed before clinical implementation.

## 1 Introduction

Head and neck cancer (HNC) is the seventh most frequently diagnosed cancer worldwide. Treatment is typically multimodal, and radiotherapy (RT) remains a cornerstone of management, being used in approximately 75% of patients, particularly in locally advanced disease (1, 2). Despite its central therapeutic role, RT is associated with a wide spectrum of acute and chronic toxicities, among which osteoradionecrosis (ORN) represents one of the most severe and debilitating chronic complications (3).

ORN of the jaw has been described as a condition caused by reduced blood supply to the bone, resulting in necrosis, with no specific duration of exposed bone (4). This condition can be detected both clinically and radiographically and is identified by sclerotic bone lesions on radiographic examination, exposed bone, or bone probeable through a periodontal pocket or fistula in previously irradiated areas, occurring at any time following ionizing radiation exposure (4, 5). Given its progressive and irreversible nature, ORN is associated with substantial morbidity and may lead to trismus, severe pain, pathologic mandibular fractures, impaired nutrition, and markedly reduced quality of life (6–8). Management remains particularly challenging due to the lack of standardized treatment protocols, often requiring complex and prolonged interventions that impose considerable economic and healthcare burdens on both patients and healthcare systems (8, 9).

Given the challenges of managing ORN, identifying high-risk patients would be beneficial. The increasing availability and storage of data and knowledge across various fields, including oral health (10), have created new opportunities to move beyond disease management to predictive and preventive care. Predictive models may support clinical decision-making by identifying high-risk patients, guiding treatment selection, and enabling targeted interventions (11). The prediction of treatment-related toxicities has traditionally relied on Normal Tissue Complication Probability (NTCP) models; however, Machine Learning (ML) and Deep Learning (DL) approaches have emerged recently as promising alternatives for this task (12, 13).

The implementation of ML in clinical settings can be challenging when datasets are small and imbalanced. This is particularly relevant for ORN, a relatively low-prevalence condition, with reported incidence ranging from 4% to 15% (14–16). Consequently, real-world ORN cohorts are inherently imbalanced, which may bias conventional algorithms toward the majority class and compromise predictive performance (17). Addressing this issue often requires specialized strategies such as data resampling or algorithmic adjustments to improve prediction of less common but clinically significant outcomes (18).

Despite this, prediction models for ORN remain scarce, particularly in South American populations. Therefore, this preliminary study aimed to develop an ML-based prediction model for ORN in HNC patients, using clinical and mandibular dose-volume data from a South American cohort. The study was conducted to explore clinically relevant predictors of ORN and to provide preliminary evidence supporting future multicenter validation and model refinement. To the best of our knowledge, this is the first ML-based ORN prediction study using a South American cohort.

## 2 Materials and methods

### 2.1 Study design

This preliminary retrospective study used routinely collected clinical and dosimetric data for model development and internal validation. The study was reported in accordance with the Transparent Reporting of a multivariable prediction model for Individual Prognosis Or Diagnosis (TRIPOD+AI) (19) (**Supplementary Table 1**). It was registered in Open Science Framework (https://doi.org/10.17605/OSF.IO/XR4SF). Ethical approval was obtained from the Ethics Committee of the Faculty of Health Sciences, University of Brasília (protocol number 72787723.4.0000.0030) and the study was conducted in accordance with the ethical principles of the Declaration of Helsinki.

Head and neck cancers were defined according to the International Classification of Diseases, 10th Revision (ICD-10) and included codes C00–C14, C30–C32, C73, and C44.3.

### 2.2 Medical data records

Data were collected from the medical records of the High-Complexity Oncology Care Center at the University Hospital of Brasília (HUB), Brazil. We included patients who underwent RT for HNC between 2019 and 2024 and completed the prescribed treatment course. We excluded patients who discontinued RT, as they did not complete the prescribed radiation course, as well as those with unavailable clinical or dosimetric data.

All patients in this cohort were treated with three-dimensional conformal radiation therapy (3D-CRT). Clinical and dosimetric data extracted from medical records were used as candidate predictors for ORN. All candidate predictors were derived exclusively from the pre-RT period, ensuring a predictive study design. ORN occurrence after RT was collected separately and used exclusively as the outcome variable; it was not included in feature selection or model development.

#### 2.2.1 Clinical data

The clinical variables collected included:

- Continuous variables: age, prescribed total radiation dose, fractionated dose per day, and the total number of fractions, smoking duration;

- Categorical variables: Tumor location, Union for International Cancer Control (UICC) (20), stage, histopathological diagnosis, Tumor (T) status, Node (N) Status, RT intent, marital status, ethnicity, level of education, smoking status, alcohol status, systemic diseases, use of medications;

- Binary variables: biological sex, chemotherapy, cancer surgery prior to RT, tooth extraction before RT, endodontic treatments before RT, scaling before RT.

Tumor location was initially recorded using multiple anatomical categories and subsequently one-hot encoded into separate binary variables. Accordingly, oral cavity tumor location was coded as 1 (yes) for oral cavity tumors and 0 (no) for all other tumor sites.

#### 2.2.2 Dosimetric data

Segmentation of the mandible, including the condyles but excluding the teeth, was performed manually by the researcher (VTC) under the supervision of a radiation oncologist (WCA), using Varian Medical Systems Treatment Planning System, Inc. (headquartered in Palo Alto, California, USA) on the treatment planning computed tomography images. The segmentation was independently reviewed for all patients; however, no interobserver variability was assessed. The mandible volume was extracted in cm³, along with the following dosimetric data: minimum dose, mean dose, maximum dose, D2%, D97%, D98%, and D99%; doses from D5% to D95% in 5% increments; and volumes from V5Grays (Gy) to V70Gy in 5 Gy increments. Dose metrics (D) were corrected to an equivalent dose in 2-Gy fractions (EQD2) to account for differences in biological effect across the fractionation schedules used in the cohort. An α/β ratio of 3 Gy was assumed to reflect late-responding normal tissue effects. Volume metrics (V) were retained in physical dose (13, 21).

### 2.3 Outcome

The primary outcome was mandibular ORN documented during the available follow-up after radiotherapy. ORN diagnoses were retrospectively obtained from patients’ medical and dental records, with follow-up extending from the completion of radiotherapy until the last available record, regardless of whether ORN was diagnosed during follow-up, and outcome ascertainment ending in August 2026. For patients who developed ORN, time to diagnosis was calculated from the completion of radiotherapy to the first documented ORN diagnosis. Patients were classified as having ORN if, at any time after radiotherapy, they had a history of mandibular irradiation together with clinical evidence of exposed bone and/or radiographic evidence of bone necrosis; no minimum duration of bone exposure was required. Outcome assessment was performed during routine clinical care, independently of the present study. No fixed prediction horizon was prespecified; therefore, the model estimated ORN occurrence during the available follow-up rather than within a specific post-radiotherapy period.

### 2.4 Data preprocessing

All categorical variables were transformed into numerical format using one-hot encoding prior to model development. This process decomposed each categorical feature into binary indicators (yes/no); thus, each category level was represented as an independent variable. Feature standardization was applied using a scaling pipeline for continuous variables for Support Vector Machines (SVM) and Logistic Regression (LR).

### 2.5 Predictor selection

A prior clinical dimensionality reduction was performed to reduce the initial set of candidate predictors by excluding variables considered not clinically relevant to ORN prediction. Variables with more than 20% missing data were subsequently excluded from the remaining candidate predictors. These two outcome-independent preliminary filtering steps were performed before cross-validation. Variables with missingness below the 20% threshold were retained for subsequent predictor screening. No imputation was performed before cross-validation.

Predictor screening and selection were performed within a nested bootstrap-within-cross-validation framework. This involved 10 repetitions of stratified 5-fold CV, in which multicollinearity screening and variable selection were performed exclusively within the training partition of each fold, using 100 bootstrap resamples, without using information from the corresponding test partition. Within each training fold, missing values were imputed using medians calculated exclusively from that training partition before bootstrap resampling. The same training-fold medians were used to impute missing values in the corresponding test partition. Median imputation was also applied to one-hot-encoded binary indicators, when necessary. Thus, no information from the test partition was used to determine the imputation values.

Multicollinearity screening was performed separately within each training fold. Pearson correlation coefficients were calculated among candidate predictors using the imputed training data. When two variables were highly correlated (*r* > 0.8), exploratory univariable LR was performed using only the training-fold outcome data, and the variable with the weaker univariable association with ORN was removed. Thus, neither the predictors nor the outcomes from the corresponding test partition informed the multicollinearity screening.

After multicollinearity screening, multivariable forward stepwise LR was performed in each bootstrap resample of the corresponding training fold. At each forward step, candidate variables not yet included in the model were evaluated using Wald p-values. The variable with the lowest p-value was selected for potential inclusion. Retention of the selected variable was confirmed only if its individual p-value was below the significance threshold (p < 0.05) and if its inclusion resulted in a decrease in the overall Akaike Information Criterion (AIC) compared to the previous model iteration. Final predictors were chosen according to their selection frequency across all 5,000 iterations. None of the predictors retained in the final models had missing values.

### 2.6 Machine learning

#### 2.6.1 Model training, validation and approaches

To identify the most robust model for predicting ORN, a comparative benchmarking of six different ML algorithms, LR, Random Forest (RF), SVM, CatBoost, Extreme Gradient Boosting (XGBoost), and Tabular Prior-Data Fitted Network (TabPFN) (22), was conducted.

A repeated stratified 5-fold cross-validation strategy was employed. This procedure was repeated 10 times, generating 50 cross-validated estimates per model. All algorithms were trained and evaluated using identical data splits across iterations, ensuring consistent comparison under the same resampling conditions. Stratification distributed the eight ORN cases as evenly as possible across the five folds, resulting in six or seven ORN cases in each training fold and one or two cases in each held-out test fold. This framework was applied under three distinct strategies: Class-weighted baseline modelling, data augmentation, and probability threshold adjustment, as described below:

- Class-weighted baseline modelling: In this approach, no data augmentation or probability threshold optimization was applied. Class-imbalance weighting was incorporated into the algorithms that supported this functionality, using balanced class weights for LR, RF, SVM, and CatBoost and scale_pos_weight for XGBoost. No additional class-weighting parameter was applied to TabPFN.

- Data augmentation (Synthetic Minority Over-sampling Technique for Nominal and Continuous features - SMOTE-NC): To address class imbalance at the data level, SMOTE-NC, a data augmentation strategy implemented via the *imbalanced-learn* library (23), was applied only to the training data within each fold. This strategy was used as an exploratory approach to address class imbalance while preserving the original distribution of the held-out test folds. Each test fold was then evaluated using a model trained on the corresponding augmented training fold. Class-imbalance weighting remained enabled in algorithms supporting this functionality, with the corresponding weights determined from the resampled training data after SMOTE-NC. This method was selected to accommodate mixed feature types (continuous and categorical) and to generate synthetic samples for the minority class (ORN cases) using five nearest neighbors (k = 5), avoiding any data leakage from the test set.

- Probability threshold adjustment based on Youden’s Index: To address class imbalance at the decision level, the classification threshold was optimized within each training fold using Youden’s Index (*J* = Sensitivity + Specificity − 1). The selected threshold was then applied to the corresponding held-out fold to generate the final classifications. This approach was intended to improve minority-class detection while avoiding information leakage from the evaluation fold.

No hyperparameter tuning or hyperparameter search was performed for any algorithm. Model hyperparameters were predefined and held fixed throughout the cross-validation procedure and modelling strategies, except for class-imbalance-related parameters, which were determined based on the corresponding training data. The complete model configurations and hyperparameters are reported in **Supplementary Table 2**, while the software environment and package versions used in the analysis are provided in **Supplementary Table 3**.

All three modelling strategies were fully evaluated and compared using identical repeated cross-validation splits. After evaluation, the strategy showing the most consistently higher internally cross-validated sensitivity and F1 score estimates across models was emphasized descriptively in the main results. This emphasis did not represent formal strategy selection within a nested cross-validation framework.

The final LR model coefficients, intercept, predictor coding, and equation for individual risk estimation are provided in **Supplementary Table 4**.

#### 2.6.2 Model performance

Model performance was primarily evaluated using sensitivity, selected because the main clinical concern in ORN risk prediction is to minimize false-negative classifications. F1 score was also reported as a key metric because of its usefulness in imbalanced scenarios and because high sensitivity may increase the number of false positives. Additional classification metrics included the area under the receiver operating characteristic curve (ROC-AUC), precision, specificity, and accuracy. Logarithmic loss (log loss) and the Brier score were used to assess the probabilistic quality of model predictions. All performance metrics were reported as mean values with 95% confidence intervals. Furthermore, decision curve analysis was performed as an exploratory assessment of potential clinical utility by calculating the net benefit across a range of threshold probabilities. Descriptive and mathematical definitions of all metrics are provided in **Supplementary Table 5**.

#### 2.6.3 Sensitivity analysis and benchmark analysis

Five sensitivity analysis and one benchmark analysis were performed to assess the robustness of the findings. First, the predictor-selection procedure was repeated using the original, unconverted physical dose metrics to assess whether EQD2 conversion influenced predictor selection. Second, model performance was reassessed using mandibular V60Gy as the sole predictor to evaluate the additional predictive contribution of oral cavity tumor location. Third, models were evaluated using oral cavity tumor location as the sole predictor to assess the contribution of V60Gy. Fourth, the analysis was repeated after excluding patients with less than one year of follow-up. This pragmatic cutoff was selected to reduce the potential impact of very limited outcome ascertainment, as ORN is most frequently diagnosed within the first few years after radiotherapy, although it may occur at any time following treatment completion (24). Finally, the analysis was restricted to patients with tumor sites considered anatomically relevant to mandibular ORN to assess the influence of tumor location on model performance. Additionally, a naive rule predicting ORN for patients with an oral cavity primary tumor was evaluated directly in the complete cohort. The same modelling and validation procedures were applied, with random oversampling replacing SMOTE-NC in the location-only analysis because its sole predictor was categorical. SMOTE-NC used four nearest neighbors (k = 4) in the tumor-site sensitivity analysis because some training folds contained only five ORN events.

### 2.7 Statistical analysis

Descriptive statistics were used to summarize the dataset, with continuous variables expressed as mean and standard deviation (SD) and categorical data presented as absolute frequencies and percentages. Normality was assessed using the Shapiro–Wilk test, followed by the Mann–Whitney U test for group comparisons. The relationship between oral cavity tumor location and mandibular V60Gy was assessed descriptively using the Mann–Whitney U test, with rank-biserial correlation used as a measure of effect size. Exploratory univariable logistic regression analysis were performed. These analyses were conducted using Jamovi (version 2.3.28). All other statistical tests, data preprocessing, and ML algorithms were implemented using Python 3.11.7. For all analyses, a p-value < 0.05 was considered statistically significant.

For exploratory comparisons of performance across the six ML algorithms and three classification strategies, pairwise comparisons of sensitivity and F1 score were performed using the Nadeau–Bengio corrected paired *t*-test. The observational unit for these comparisons was the performance estimate obtained from each cross-validation split. Results from the 50 repeated cross-validation splits were not treated as independent observations; instead, the Nadeau–Bengio correction, incorporating the test-to-training sample size ratio, was applied to the standard error of the paired differences to account for the dependence induced by repeated and overlapping training samples. Comparisons were paired using results obtained from the same cross-validation split (same repeat and fold). Holm correction was applied across all pairwise comparisons within each performance metric to control for multiple testing. Performance metrics were reported as mean values with 95% CIs. For performance metrics bounded between 0 and 1, CIs were calculated on the arcsine-square-root transformed scale. The Nadeau–Bengio correction was applied to the standard error on the transformed scale to account for dependence among the 50 repeated cross-validation splits, using a test-to-training sample size ratio of 1/4 for five-fold cross-validation. The resulting 95% CIs were then back-transformed to the original scale. When all fold-level estimates were identical at a boundary (0 or 1), the CI was considered not estimable (NE). These intervals characterize variability across repeated cross-validation splits rather than conventional population-level uncertainty. Calibration intercepts and slopes were estimated using patient-level aggregated out-of-fold predicted probabilities, with 95% confidence intervals obtained by patient-level bootstrap resampling. Calibration curves with 95% bootstrap confidence bands were constructed using the same patient-level aggregated predictions.

Figures were created using the Canva platform (canva.com), Microsoft PowerPoint (Microsoft 365, v. 2603), Python (via Spyder IDE in the Anaconda distribution) and Google Gemini 3 Flash to generate illustrative icons.

## 3 Results

### 3.1 Patient cohort and baseline characteristics

The initial dataset comprised 117 patients. After excluding those with missing dose-volume histogram data (n=6), patients who did not complete treatment due to death or clinical deterioration (n=12), and those with unavailable medical records (n=2), a total of 97 patients were included in the analysis, among whom 8 (8.25%) developed ORN (**Figure 1**). Across the repeated cross-validation procedure, each training fold included approximately 77–78 patients, including six or seven ORN cases, whereas each held-out test fold included 19–20 patients, including one or two ORN cases. The median time from radiotherapy completion to ORN diagnosis was 186 days (range: 8–1,287 days). In this cohort, no patient developed ORN outside of the mandible.

**Figure 1:**
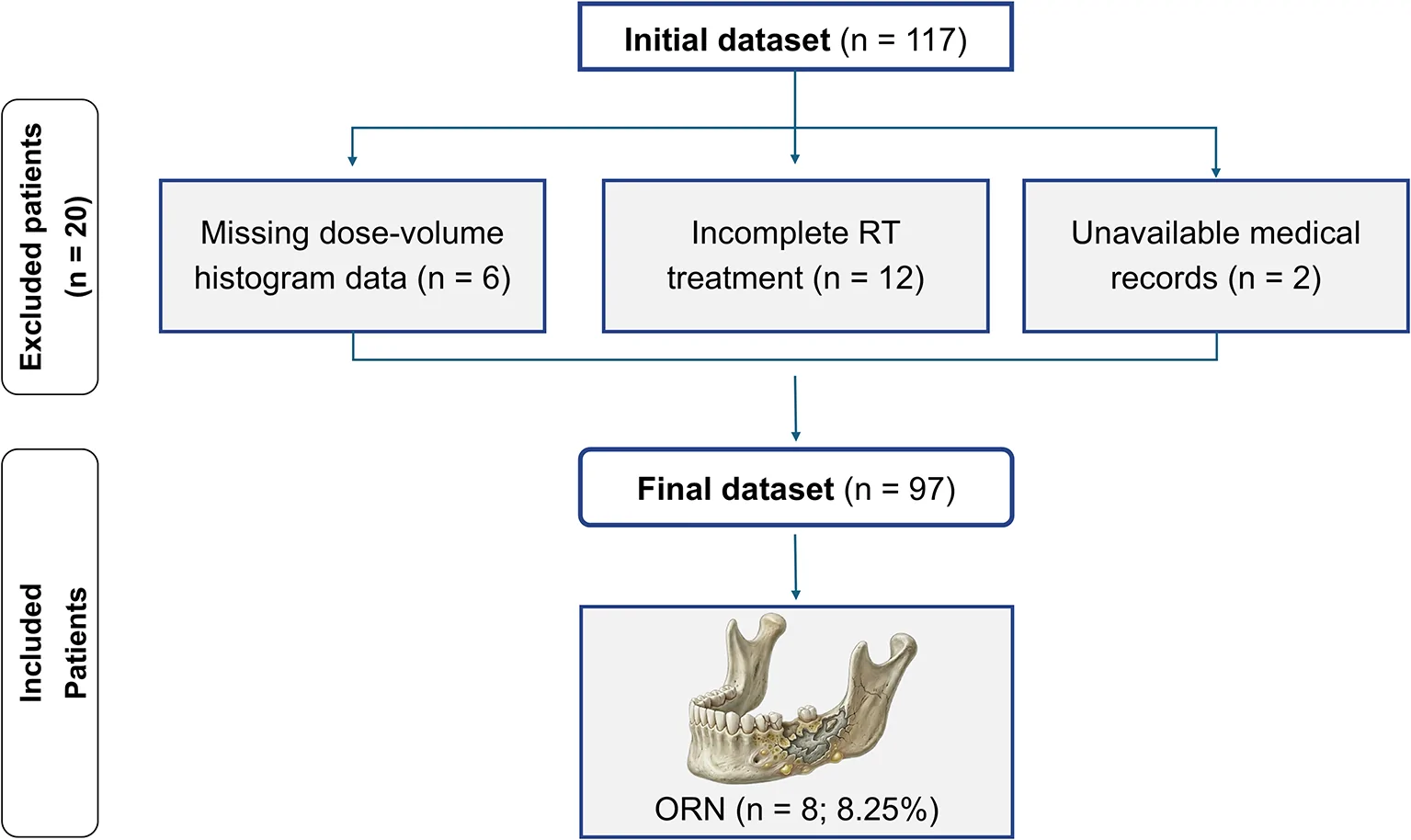
Flowchart of patient selection and study cohort recruitment. The diagram illustrates the exclusion criteria applied to the initial dataset (n = 117), the final distribution of the study population (n=97) and the incidence of osteoradionecrosis in the study cohort (8.25%). n, number of patients; ORN, osteoradionecrosis; RT, radiotherapy.

Follow-up duration was calculated from the completion of radiotherapy to the last available clinical assessment. The median follow-up duration was 23.28 months (interquartile range [IQR]: 12.46–37.94; range: 1.91–84.89 months). Among patients who developed ORN, the median follow-up duration was 39.98 months (IQR: 33.34–53.84; range: 12.46–84.89 months), whereas among patients without ORN, it was 21.83 months (IQR: 11.27–32.20; range: 1.91–84.49 months).

The mean age of the cohort was 62.6 years (SD 13.83), and most of the study population was male (85.57%). The cohort mainly included patients with laryngeal (35.05%), oropharyngeal (23.71%), and oral cavity (21.65%) cancers (**Table 1**). Advanced disease was prevalent, with over 60% of patients presenting with T3 (29.9%) or T4 status (31.96%) and 50.5% having nodal involvement (N1–N3). Most participants were former or current smokers (79.38%) and had a history of alcohol consumption (68.04%). In terms of treatment, 67.01% of the population received chemotherapy, 20.62% underwent surgery prior to RT, and 25.77% required tooth extractions before the start of treatment. The mean prescribed total radiation dose at the tumor site was 64.33 Gy (SD 12.45), and the mean mandibular dose was 37.33 Gy (SD 21.62). Missing data varied across demographic and staging variables. The highest proportions of missing data were observed for education level (64.95%), histological grade (31.96%), involved regional lymph nodes (27.84%), marital status (25.77%), and Tumor M status (25.77%). Missingness was lower for alcohol status (14.43%), smoking status (11.34%), T status (10.31%), cancer stage (7.22%), N status (7.22%), and ethnicity (3.09%). Comprehensive details on all patient variables are provided in the **Supplementary Tables**: **S6** (Demographics), **S7** (Clinical and Lifestyle), **S8** (Tumor and Treatment Characteristics), **S9** (Oral Health Interventions), and **S10** (Mandible Dose–Volume Histogram Parameters).

**Table 1:** Demographic and clinical characteristics of the study population.

Variable	Category	n	Percentage (%)
Age (mean, years)	–	62.6 (SD: 13.83)	–
Smoking duration (mean, years)	–	34.64 (SD: 17.92)	–
Prescribed total radiation dose (Gy) (mean)	–	64.33 (SD: 12.45)	–
Mean dose in mandible (Gy)	–	37.33 (SD: 21.62)	–
Sex	Female	14	14.43
	Male	83	85.57
Tumor location	Larynx	34	35.05
	Oral Cavity	21	21.65
	Oropharynx	23	23.71
	Nasopharynx	6	6.19
	Hypopharynx	1	1.03
	Salivary Glands	6	6.19
	Thyroid	2	2.06
	Nasal Cavity And Paranasal Sinuses	1	1.03
	Ear	1	1.03
	Unspecified primary site*	2	2.06
T status	T1	14	14.43
	T2	13	13.40
	T3	29	29.90
	T4	31	31.96
	NA	10	10.31
N status	N0	41	42.27
	N1	7	7.22
	N2	34	35.05
	N3	8	8.25
	NA	7	7.22
Smoking status	Current smoker	29	29.90
	Former smoker	48	49.48
	Never smoker	9	9.28
	NA	11	11.34
Alcohol status	Current	20	20.62
	Former	46	47.42
	Never	17	17.53
	NA	14	14.43
Chemotherapy	Yes	65	67.01
	No	25	25.77
Cancer surgery before RT	Yes	20	20.62
Tooth extraction before RT	Yes	25	25.77

*The two patients with an unspecified primary site were coded as 0 (not oral cavity) for the binary oral cavity tumor location indicator.

Gy, Gray (unit of absorbed radiation dose); n, Number of patients; N, Nodal status (TNM staging); NA, Not available; RT, Radiotherapy; SD, Standard Deviation; T, Tumor status (TNM staging); % – Percentage.

### 3.2 Predictor selection

From the initial 80 candidate features, 11 were excluded during the prior clinical dimensionality-reduction step, and 4 were excluded because they had more than 20% missing data, leaving 65 candidate features. Multicollinearity screening was subsequently performed separately within each training fold; therefore, the number of features retained varied across folds. In the resampling-based forward stepwise selection procedure, oral cavity tumor location and mandibular V60Gy had the highest selection frequencies, being selected in 43.48% and 39.02% of the 5,000 iterations, respectively. Oral cavity tumor location survived multicollinearity screening in all 50 training folds, whereas V60Gy survived in 40 folds. The variables excluded during the preliminary filtering steps are detailed in **Supplementary Table 11**, and the selection frequencies and multicollinearity-screening results for the six most frequently selected variables are reported in **Supplementary Table 12**. Given the small number of ORN events, the final predictor set was intentionally restricted to the two most frequently selected variables to reduce overfitting and preserve interpretability. These predictors were subsequently used to train and internally validate all models (**Figure 2**).

**Figure 2:**
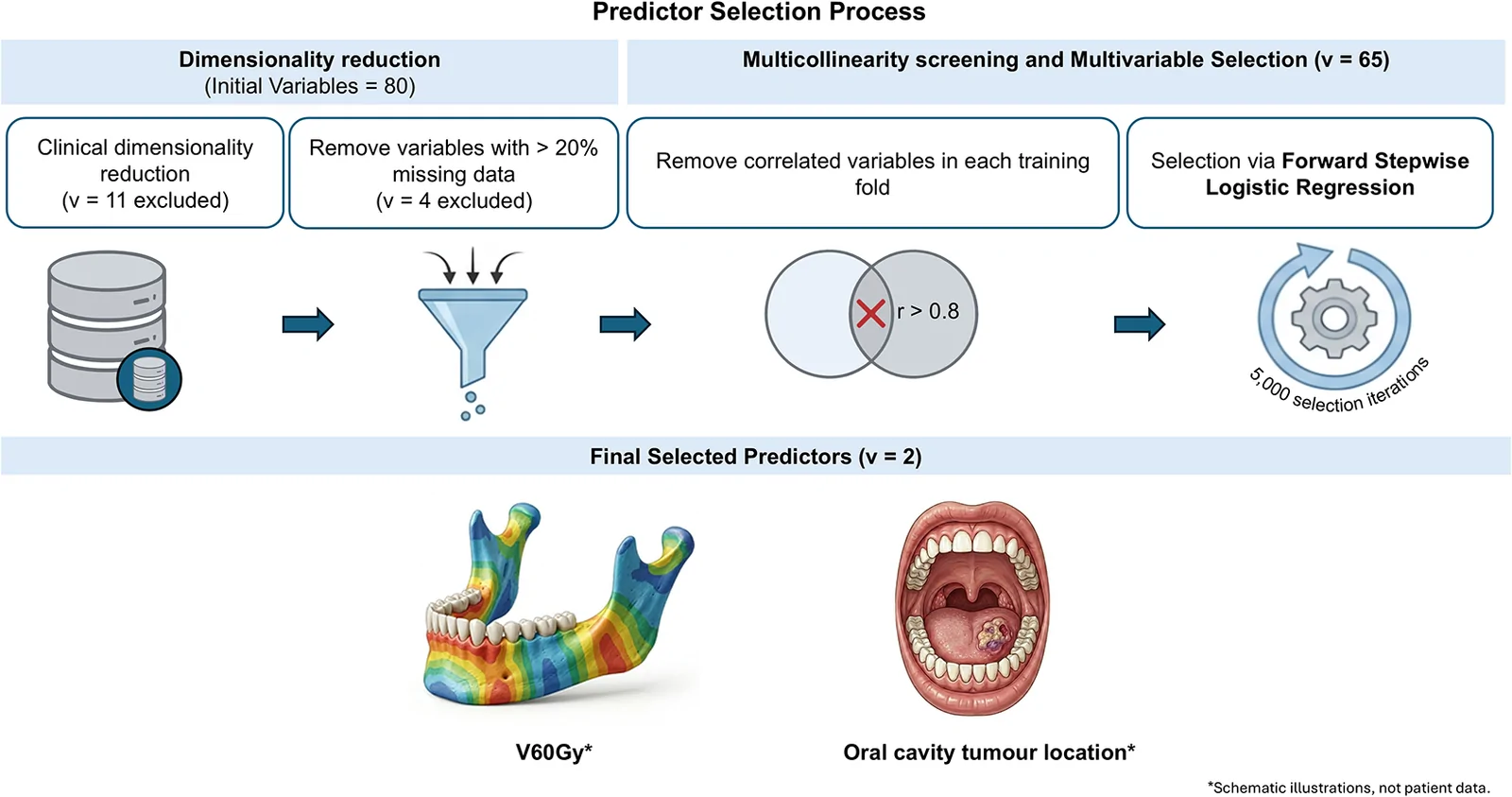
Feature selection workflow. This process began with 80 candidate predictors. A prior clinical dimensionality-reduction step excluded 11 variables, followed by the exclusion of 4 variables with >20% missing data. The remaining 65 variables underwent multicollinearity screening separately within each training fold (Pearson correlation, *r* > 0.8); therefore, the number of retained variables varied across folds. Multivariable forward stepwise logistic regression was subsequently performed within the bootstrap resamples of each training fold. Predictor selection was performed through 5,000 selection iterations using a nested bootstrap-within-cross-validation framework (10 repetitions of 5-fold cross-validation with 100 bootstrap resamples per training fold). This process identified two final predictors: mandibular V60Gy and oral cavity tumor location. The mandibular and intraoral graphics are AI-generated schematic illustrations and are not derived from patient data or actual dose distributions. Gy, Gray; LR, Logistic Regression; ML, Machine Learning; v, number of variables; V60Gy, percentage of mandibular volume receiving ≥60 Gy.

As mandibular V60Gy was retained as a final candidate predictor, group comparisons were performed using the Mann–Whitney U test. Mandibular V60Gy was higher in patients who developed ORN (median 69.6% [19.6–89.6]) than in those without ORN (median 15.3% [0.0–93.6], p = 0.001). Because V60Gy was selected based on its association with the outcome, this post-selection p-value is presented descriptively and should not be interpreted as confirmatory inference.

### 3.3 Model performance and calibration

In the class-weighted baseline approach, most models achieved high specificity and accuracy but showed limited sensitivity for the minority class. This pattern indicates that several algorithms tended to favor the majority class, correctly classifying non-ORN cases while failing to detect many ORN events. Logistic regression showed the highest sensitivity among the class-weighted baseline models, with a sensitivity of 0.74 (95% CI: 0.40–1.00) and an F1 score of 0.42 (95% CI: 0.19–0.64). In contrast, SVM, TabPFN, XGBoost and RF showed very low sensitivity under the class-weighted baseline strategy, despite apparently high accuracy and specificity.

Among the evaluated strategies, data augmentation showed the most consistently higher internally cross-validated sensitivity and F1 score estimates across models and was therefore emphasized descriptively in the main results. Complete results for all strategies are reported in **Supplementary Table 13**.

With data augmentation, cross-validated sensitivity ranged from 0.72 to 0.77 across models, and specificity from 0.83 to 0.86 (**Figure 3A**, **Table 2**). This strategy led to overall improvements in F1 scores, which reached up to 0.48 for the TabPFN model (95% CI: 0.23–0.72) and 0.47 for CatBoost (95% CI: 0.23–0.72). The ROC curves were consistent with these findings (**Figure 4A**), with LR (AUC = 0.87; 95% CI: 0.72–1.00) and TabPFN (AUC = 0.85; 95% CI: 0.72–0.98) showing the highest AUC values among the augmented models (**Table 2**). Although data augmentation resulted in slightly worse calibration metrics compared to the class-weighted baseline approach for some models, calibration estimates showed substantial uncertainty, with deviations from ideal calibration. Complete calibration results are reported in **Table 2** and **Supplementary Table 13**. No statistically significant differences in sensitivity or F1 score were observed among the models within the data augmentation approach. Compared with the class-weighted baseline strategy, data augmentation yielded higher sensitivity for SVM (Holm-adjusted p = 0.0047) and TabPFN (Holm-adjusted p = 0.0164), as well as a higher F1 score for SVM (Holm-adjusted p = 0.0431). Additional pairwise comparisons and Holm-adjusted p-values are reported in **Supplementary Tables S14**, **S15**.

**Figure 3:**
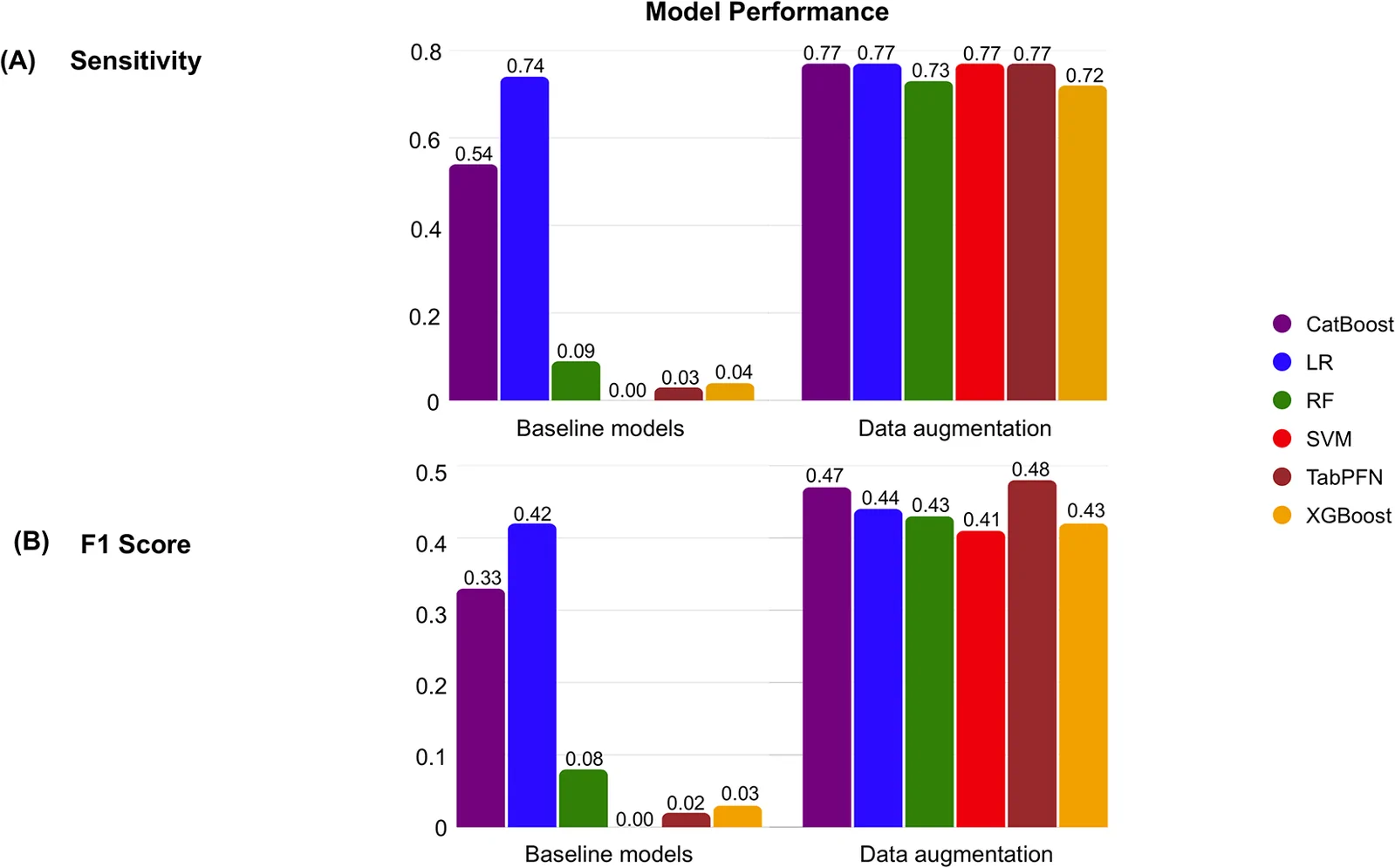
Comparison of model performance. **(A)** Comparison of sensitivity between class-weighted baseline modelling and data augmentation. **(B)** Comparison of F1 score between class-weighted baseline modelling and data augmentation.

**Table 2:** Model performance under the class-weighted baseline, threshold-adjustment, and data-augmentation strategies (mean and 95% CI). .

Approach	Model	AUC	Sensitivity	Specificity	F1 Score	Brier
Class-weighted baseline models	CatBoost	0.82 (0.66–0.97)	0.54 (0.04–1.00)	0.86 (0.75–0.97)	0.33 (0.02–0.65)	0.13 (0.06–0.19)
	LR	0.87 (0.74–0.99)	0.74 (0.35–1.00)	0.83 (0.72–0.95)	0.42 (0.13–0.71)	0.15 (0.09–0.21)
	RF	0.73 (0.55–0.90)	0.09 (0.00–0.23)	0.96 (0.92–1.00)	0.08 (0.00–0.17)	0.09 (0.06–0.12)
	SVM	0.85 (0.69–0.99)	0.00 (NE)	0.999 (0.998–1.000)	0.00 (NE)	0.07 (0.05–0.09)
	TabPFN (Unweighted)	0.85 (0.72–0.97)	0.03 (0.00–0.09)	0.98 (0.96–1.00)	0.02 (0.00–0.05)	0.07 (0.05–0.10)
	XGBoost	0.78 (0.65–0.91)	0.04 (0.00–0.08)	0.94 (0.87–1.00)	0.03 (0.00–0.06)	0.12 (0.06–0.17)
Data augmentation	CatBoost	0.84 (0.67–0.99)	0.77 (0.40–1.00)	0.86 (0.76–0.96)	0.47 (0.16–0.79)	0.12 (0.05–0.20)
	LR	0.87 (0.73–0.99)	0.77 (0.40–1.00)	0.84 (0.74–0.94)	0.44 (0.16–0.71)	0.13 (0.06–0.20)
	RF	0.84 (0.67–0.99)	0.73 (0.27–1.00)	0.86 (0.77–0.95)	0.43 (0.11–0.74)	0.13 (0.06–0.21)
	SVM	0.84 (0.68–0.99)	0.77 (0.40–1.00)	0.83 (0.74–0.93)	0.41 (0.15–0.68)	0.13 (0.07–0.20)
	TabPFN	0.85 (0.71–0.98)	0.77 (0.40–1.00)	0.86 (0.76–0.96)	0.48 (0.16–0.80)	0.13 (0.05–0.20)
	XGBoost	0.82 (0.63–0.99)	0.72 (0.24–1.00)	0.86 (0.75–0.97)	0.43 (0.08–0.79)	0.14 (0.05–0.22)
Threshold adjustment	CatBoost	0.82 (0.66–0.97)	0.41 (0.00–0.95)	0.90 (0.80–0.99)	0.27 (0.00–0.59)	0.13 (0.06–0.19)
	LR	0.87 (0.74–0.99)	0.63 (0.12–1.00)	0.87 (0.77–0.97)	0.39 (0.05–0.73)	0.15 (0.09–0.21)
	RF	0.73 (0.55–0.90)	0.09 (0.00–0.23)	0.97 (0.93–1.00)	0.08 (0.00–0.17)	0.09 (0.06–0.12)
	SVM	0.85 (0.69–0.99)	0.65 (0.15–1.00)	0.85 (0.75–0.95)	0.38 (0.07–0.68)	0.07 (0.05–0.09)
	TabPFN	0.85 (0.72–0.97)	0.66 (0.10–1.00)	0.85 (0.73–0.97)	0.37 (0.03–0.72)	0.07 (0.05–0.10)
	XGBoost	0.78 (0.65–0.91)	0.04 (0.00–0.08)	0.95 (0.89–1.00)	0.03 (0.00–0.07)	0.12 (0.06–0.17)

Class weighting was applied to all baseline models except TabPFN, which was evaluated without class weighting. Performance metrics, including F1 score, were calculated separately for each cross-validation split and subsequently averaged across the 50 splits; they were not calculated from pooled confusion-matrix counts. Confidence intervals were calculated on the arcsine-square-root-transformed scale using the Nadeau–Bengio correction with a test-to-training sample-size ratio of 1/4 and were subsequently back-transformed to the original scale. NE indicates that the confidence interval was not estimable because all fold-level estimates were identical at the boundary. These intervals characterize variability across repeated cross-validation splits rather than population-level uncertainty.

AUC, area under the receiver operating characteristic curve; Brier, Brier score; CatBoost, Categorical Boosting; CI, confidence interval; LR, Logistic Regression; NE, not estimable; RF, Random Forest; SVM, Support Vector Machine; TabPFN, Tabular Prior-Data Fitted Network; XGBoost, Extreme Gradient Boosting.

**Figure 4:**
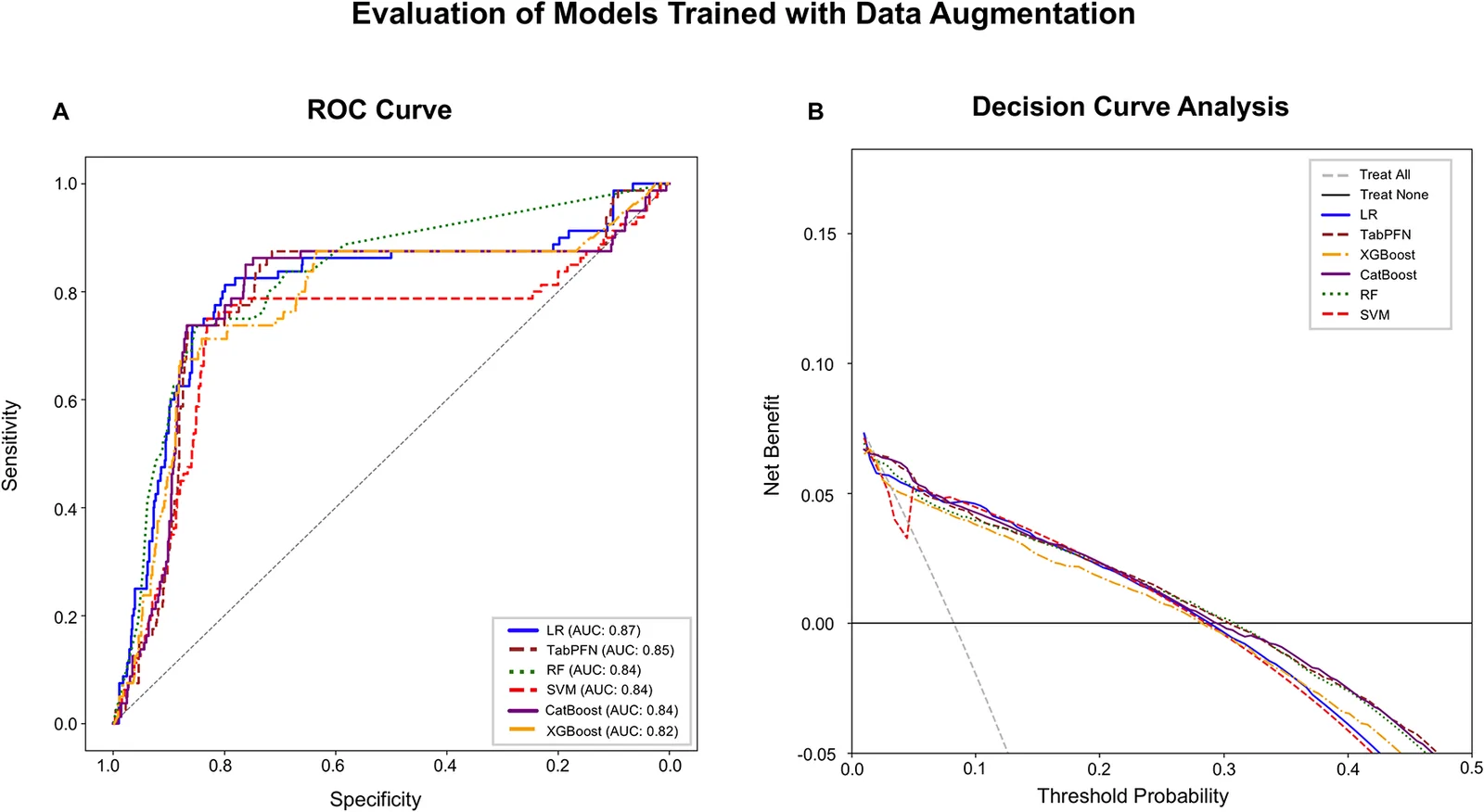
Evaluation of models trained with data augmentation. **(A)** ROC curves showing the discriminative performance of the models, with LR achieving the highest AUC (0.87). **(B)** Exploratory Decision Curve Analysis (DCA) showing the net benefit of the augmented models across threshold probabilities. Positive net benefit was observed at lower threshold probabilities, particularly below approximately 30%; however, no intervention-specific threshold was prespecified. ROC and decision curves were constructed using pooled out-of-fold predictions across the repeated cross-validation splits. AUC values shown in the legend represent the mean of the fold-level AUC estimates reported in **Table 2** rather than the AUC calculated from the pooled ROC curve. AUC, area under the receiver operating characteristic curve; Brier, Brier score; CatBoost, Categorical Boosting; CI, confidence interval; LR, Logistic Regression; NE, not estimable; RF, Random Forest; SVM, Support Vector Machine; TabPFN, Tabular Prior-Data Fitted Network; XGBoost, Extreme Gradient Boosting.

Given the small number of ORN events, absolute classification counts across repeated out-of-fold (OOF) predictions were also examined. Across the 10 cross-validation repetitions, the eight unique ORN cases contributed 80 OOF predictions. With data augmentation, the models correctly classified 54–59 of these 80 ORN predictions (true positives - TP), while 21–26 were classified as false negatives (FN). Corresponding pooled PPV values ranged from 0.28 to 0.33. These counts represent repeated OOF predictions rather than independent patients and are reported to provide additional context for the performance estimates (**Supplementary Table 16**).

The comparison based on F1 score revealed marked differences across approaches. Under class-weighted baseline modelling, LR retained measurable minority-class performance (F1 = 0.42), whereas several other models showed limited performance, with low F1 scores. Data augmentation improved F1 scores across all evaluated algorithms. This strategy resulted in higher F1 scores across all models and allowed TabPFN and CatBoost to achieve the highest F1 scores observed in the study (0.48 and 0.47, respectively) (**Figure 3B**).

Exploratory decision-curve analysis (**Figure 4B**) showed positive net benefit for the augmented models at threshold probabilities below approximately 30%. However, because no intervention-specific threshold probability was prespecified, these findings should not be interpreted as evidence of established clinical utility. Calibration plots based on patient-level aggregated out-of-fold predictions, with 95% bootstrap confidence bands, showed substantial uncertainty, particularly under class-weighted baseline modelling. With data augmentation, the calibration curves were more similar across models, and the uncertainty bands were generally narrower, although deviations from the ideal calibration line remained. Calibration intercept and slope estimates also showed considerable uncertainty, with wide confidence intervals for several models. Given the small number of ORN events, calibration estimates should be interpreted cautiously (**Supplementary Figure 17**; **Supplementary Table 18**). Comprehensive details for all algorithms, including all class-imbalance strategies, calibration, performance metrics, error metrics, and optimized threshold values, are provided in **Supplementary Table 13**.

### 3.4 Sensitivity analysis and benchmark analysis

After excluding patients with less than one year of follow-up, 75 patients remained, including all 8 patients who developed ORN. The overall pattern of model performance remained consistent with the primary analysis. Sensitivity with data augmentation ranged from 0.65 to 0.73, while F1 scores ranged from 0.40 to 0.50. Performance estimates across all evaluated metrics remained within the corresponding 95% confidence intervals from the primary analysis, with no substantial changes in the overall interpretation of model performance (**Supplementary Table 19**).

In the sensitivity analysis restricted to tumor sites considered most anatomically relevant to mandibular ORN (oral cavity and oropharynx), 44 patients were included, comprising 21 patients with oral cavity tumors and 23 with oropharyngeal tumors, of whom 7 developed ORN. Overall, model performance was lower than in the primary analysis. With data augmentation, sensitivity ranged from 0.42 to 0.68 and F1 scores from 0.28 to 0.41. LR achieved the highest sensitivity (0.68; 95% CI: 0.24–1.00) and F1 score (0.41; 95% CI: 0.13–0.70), although the confidence intervals were wide, reflecting the small number of events in this restricted sample. Complete results are reported in **Supplementary Table 20**.

A descriptive comparison showed that mandibular V60Gy was higher among patients with oral cavity tumors than among those with tumors at other anatomical sites (median: 64.9% vs. 3.74%; Mann–Whitney U = 274, nominal p < 0.001), with a rank-biserial association of r_rβ_ = 0.657. Given this association, an additional sensitivity analysis was performed to assess whether oral cavity tumor location provided predictive information beyond V60Gy. When oral cavity tumor location was removed as the second predictor, leaving mandibular V60Gy as the sole predictor, model performance was generally lower than in the primary analysis. With data augmentation, sensitivity ranged from 0.54 to 0.74 and F1 scores from 0.23 to 0.30, compared with 0.72–0.77 and 0.41–0.48, respectively, in the primary analysis. These findings suggest that oral cavity tumor location may provide additional predictive information beyond V60Gy alone (**Supplementary Table 21**).

Conversely, when oral cavity tumor location was used as the sole predictor, all models with random oversampling achieved a sensitivity of 0.78, specificity of 0.83, F1 score of 0.43, and AUC of approximately 0.81 (**Supplementary Table 22**). These results were like those of the naive location-based rule, which yielded a sensitivity of 0.75, specificity of 0.83, F1 score of 0.41, and AUC of 0.79 (**Supplementary Table 23**). In comparison, the full models incorporating both oral cavity tumor location and V60Gy achieved F1 scores ranging from 0.41 to 0.48 and AUCs ranging from 0.82 to 0.87 with data augmentation. Thus, adding V60Gy produced modest numerical improvements in the maximum F1 score and AUC, although improvements were not consistent across metrics or algorithms.

In the sensitivity analysis using the original, unconverted physical dose values for all dosimetric variables, the predictor-selection procedure yielded the same final predictors as the primary analysis: mandibular V60Gy and oral cavity tumor location. Therefore, EQD2 conversion of the dose-based DVH metrics did not affect the final predictor selection.

## 4 Discussion

This preliminary single-center study evaluated exploratory ML-based prediction models for ORN using clinical and mandibular dose-volume data from a real-world South American cohort. Within this cohort and internal validation framework, the models demonstrated exploratory potential for ORN risk assessment, while data augmentation resulted in higher internally cross-validated sensitivity and F1 score estimates than class-weighted baseline training strategies, suggesting improved handling of class imbalance, particularly for detecting the minority class.

The incidence (8.25%) and time to ORN development in this cohort are consistent with previously reported rates in the literature (4, 14–16). Mandibular V60Gy and oral cavity tumor location were selected as final candidate predictors, both of which are clinically relevant and readily available in radiotherapy planning. Higher radiation exposure to the mandible has been previously associated with increased ORN risk (25, 26). Previous studies have also reported that most patients who develop ORN receive doses exceeding 60 Gy at the affected site, providing clinical context for the selection of V60Gy in the present cohort (27, 28). Anatomical sites are also relevant in this context, as tumors in the oral cavity often require higher mandibular radiation doses compared to other regions (29, 30). These associations are consistent with known mechanisms of radiation-induced bone injury, characterized by impaired bone remodelling and reduced regenerative capacity driven by cellular damage and altered osteoblastic and osteoclastic activity (31). Overall, these findings indicate that the selected predictors are biologically plausible and clinically relevant. Although oral cavity tumor location was strongly associated with V60Gy, the sensitivity analysis using V60Gy as the sole predictor showed generally lower model performance, suggesting that tumor location may provide additional predictive information beyond mandibular dose exposure alone. In the reciprocal analysis, models using oral cavity tumor location alone performed similarly to the naive location-based rule and nearly as well as the full two-predictor models. Adding V60Gy yielded modest improvements in F1 score and AUC, which were inconsistent across metrics and algorithms, suggesting that oral cavity tumor location alone may capture much of the predictive information.

The selection of only two predictors also highlights the parsimonious nature of the proposed model. This finding is particularly relevant in the context of ORN prediction, where the limited number of events increases the risk of overfitting when more complex models are developed. Parsimonious models may therefore offer advantages in terms of interpretability and availability (32). Furthermore, because both predictors are routinely available during radiotherapy planning, their use minimizes data collection burden and may facilitate future implementation in clinical practice.

The target population of this study comprised patients who completed the prescribed radiotherapy course. The earliest ORN diagnosis occurred eight days after radiotherapy completion. This patient had received radiotherapy over approximately eight weeks, and ORN was diagnosed eight days after the final fraction. Current diagnostic criteria do not require a minimum latency period after radiotherapy. The patient had also undergone mandibular surgery approximately three months before starting radiotherapy. Although no pre-existing osteonecrosis was documented in the available records, this prior surgical intervention may have contributed to the early presentation. Patients who died or clinically deteriorated before treatment completion did not receive a radiation exposure comparable to that of the included patients and could not be adequately assessed for post-treatment ORN. Their inclusion could therefore have introduced exposure and outcome misclassification. Accordingly, the findings apply to patients who complete radiotherapy and are not intended to be generalized to those who discontinue treatment because of death or clinical deterioration. The applicability of our findings is further defined by the exclusive use of 3D-CRT in this cohort. Therefore, the results cannot be directly extrapolated to patients treated with contemporary techniques such as IMRT or VMAT, particularly because mandibular dose–volume distributions may vary according to treatment and planning practices. Overall, our findings are specific to the institutional, treatment, and population context represented by this cohort, requiring validation in populations treated with modern radiotherapy techniques.

Considering differences in radiotherapy techniques, cohort characteristics, predictor selection, and validation strategies, comparisons with previous studies should be interpreted cautiously. Reber et al. (33) reported F1 scores between 0.21 and 0.39, comparable to the class-weighted baseline results observed in the present study. After data augmentation, cross-validated F1 scores increased to 0.41–0.48, suggesting that class imbalance handling may facilitate prediction of this low-prevalence outcome in this cohort, rather than indicating superior model performance.

Similarly, the present study achieved AUC values ranging from 0.73 to 0.87, compared with 0.69–0.74 reported by Reber et al. (33) and 0.67–0.69 by Humbert-Vidan et al. (16), despite both studies including substantially larger cohorts. These findings do not indicate superior model performance but rather suggest that data heterogeneity and cohort composition may influence predictive performance, with more heterogeneous datasets potentially posing greater challenges for discrimination. These observations highlight the continued importance of single-center evaluations, as many clinical centers rely on limited local datasets, and differences in patient populations and treatment protocols across institutions may affect the performance of externally developed models (34, 35).

Performance varied according to the metric considered, and no algorithm consistently outperformed the others across all measures. Among the top-performing models, TabPFN, CatBoost and LR with data augmentation achieved higher results. In contrast, RF, XGBoost, TabPFN, and SVM performed poorly under class-weighted baseline modelling, likely reflecting the difficulty of learning minority class patterns from limited data (36). In practical clinical terms, higher sensitivity and F1 score after data augmentation could refine the models’ detection of patients who developed ORN in this small cohort. This increase in sensitivity is clinically relevant because reducing false negatives is essential in a low prevalence but potentially severe condition, where missed cases may delay preventive measures and compromise patient management. However, the improvement in sensitivity was accompanied by a limited F1 score, with a maximum of 0.48, suggesting that false-positive classifications remain an important consideration. In clinical practice, incorrectly identifying patients as being at high risk for ORN may lead to additional monitoring, imaging, specialist consultations, and preventive interventions, increasing healthcare resource use and costs. It may also increase patient anxiety by creating concerns about the development of a potentially serious complication. Therefore, although prioritizing sensitivity may reduce missed ORN cases, the potential clinical and psychosocial burden of false-positive predictions should be considered when defining appropriate decision thresholds.

LR showed relatively consistent discrimination and minority-class performance across the evaluated approaches, suggesting some resilience to class imbalance. This likely reflects the low-dimensional feature space and linear relationship between predictors and outcome, conditions under which LR can match or outperform more complex ML models (37), while also being less prone to overfitting in small imbalanced datasets (36). Given that the final feature set comprised only mandibular V60Gy and oral cavity tumor location, the limited dimensionality may also reduce the potential benefit of greater algorithmic complexity. Calibration estimates showed substantial uncertainty across models, precluding conclusions regarding the superiority of any algorithm in terms of probability estimation. Accordingly, the present findings do not demonstrate superiority of complex ML algorithms over conventional logistic regression. Instead, they suggest that a parsimonious two-variable logistic model may provide competitive performance with greater interpretability, although its predictive performance requires external validation.

The improvement observed after data augmentation should be interpreted primarily as an internal cross-validation finding rather than evidence of improved external generalization. SMOTE-NC is a technique that balances datasets by creating new minority-class examples from similar existing cases, blending nearby numerical values or using the most common category among the most similar cases for categorical features (23). Because synthetic minority samples were generated from few positive cases within each training fold, the diversity of minority-class patterns available to the algorithms remained inherently limited. Thus, particularly for more complex algorithms, the observed performance may reflect patterns derived from this restricted set of cases rather than generalizable relationships. The results therefore mainly show that imbalance-handling strategies can reduce majority-class bias during model development. External validation is required to determine whether this gain in sensitivity is maintained in independent patients.

Probability threshold adjustment provided a simpler alternative to data augmentation because it does not modify the training data or generate synthetic observations. However, its effect on sensitivity was model-dependent: sensitivity increased substantially for SVM and TabPFN (from 0.00 to 0.65 and from 0.03 to 0.66, respectively) but decreased for CatBoost and LR and showed little change for RF and XGBoost. In contrast, data augmentation produced more consistent sensitivity estimates across models (0.72–0.77). Therefore, although threshold adjustment may be preferable when a conservative approach is desired, particularly in small datasets where synthetic observations may introduce additional uncertainty, it did not provide a consistent sensitivity advantage in the present study. Both strategies should therefore be considered exploratory, and their relative clinical utility and external generalizability should be assessed in larger independent cohorts.

The lower performance in the sensitivity analysis restricted to patients with oral cavity and oropharyngeal tumors raises the possibility that discrimination in the full cohort may have been partly influenced by the inclusion of patients with anatomically distant tumors and minimal mandibular radiation exposure. This finding suggests that model discrimination could differ among patients with tumor sites more directly associated with mandibular irradiation.

This study has several limitations. First, the small number of ORN events represents a major limitation. With eight ORN events and two predictors, the events-per-variable ratio was 4, below the conventional minimum of 10, indicating an increased risk of overfitting and unstable model estimates. As each held-out fold contained only one or two ORN cases, sensitivity could change substantially with a single correct or incorrect classification, while precision was similarly sensitive to small variations in the numbers of true- and false-positive predictions. Therefore, both metrics are expected to be unstable and should be interpreted cautiously. Although repeated cross-validation allowed efficient use of the available data, it cannot fully overcome the uncertainty associated with low-prevalence outcomes. In addition, the confidence intervals derived from repeated cross-validation reflect internal resampling variability and should not be interpreted as evidence of external generalizability. The single-center design also limits the representativeness of the cohort and may restrict the applicability of the models to populations treated under different clinical, oral healthcare, and radiotherapy protocols. The cohort also included heterogeneous primary tumor sites, some of which are less directly associated with mandibular irradiation. However, a sensitivity analysis restricted to oral cavity and oropharyngeal tumors showed a broadly similar pattern, although performance was lower and uncertainty increased because of the reduced sample size. Another limitation is that mandibular segmentation was performed by a single evaluator, and interobserver variability was not assessed. The use of SMOTE-NC may have contributed to the observed increase in sensitivity during cross-validation; however, its impact on model performance in independent cohorts remains uncertain. Because class-imbalance handling differed across algorithms, this benchmark should not be interpreted as a comparison of their relative algorithmic capabilities. The emphasis on data augmentation was determined *post hoc* based on the observed cross-validated performance of the three classification strategies. Moreover, as this retrospective study is based on medical record data, it is subject to potential information and selection bias inherent to routine clinical documentation. Additionally, no predefined prediction horizon or minimum follow-up duration was established for patients without ORN. Follow-up may have been shortened because some patients returned to their referring healthcare units after completing radiotherapy, and subsequent diagnoses made outside the study center may not have been captured in its medical records. Consequently, patients with shorter follow-up may have been classified as non-events despite remaining at risk of subsequently developing ORN, potentially introducing outcome misclassification and censoring bias. In addition, mortality was not modelled as a competing event. Patients who died before developing ORN were classified as non-events, although death precluded their subsequent development of ORN, which may have biased the estimated ORN risk and model performance. However, a sensitivity analysis excluding patients with less than one year of follow-up yielded results consistent with the primary analysis, providing some support for the robustness of the findings to shorter follow-up. Finally, the restricted number of predictors included in the final model, while improving interpretability and reducing overfitting, may have excluded additional clinically relevant variables that could further enhance predictive performance.

Despite these limitations, this study has notable strengths. It used a real-world clinical cohort with systematically collected clinical and dosimetric data, as well as ORN cases within the reported prevalence, thereby supporting clinical relevance. Multiple ML algorithms were evaluated under a rigorous repeated cross-validation framework with different class imbalance strategies, enabling robust model comparison. Performance assessment also extended beyond discrimination to include calibration and exploratory decision-curve analysis, providing a more comprehensive evaluation. Finally, the focus on a low-prevalence yet clinically significant outcome, combined with an interpretable and simple model, based on only two routinely available predictors, supports its potential utility for future ORN risk-stratification studies.

## 5 Conclusion

In this preliminary single-center study using a real-world South American cohort, ML-based models demonstrated exploratory potential for ORN risk assessment within an internal validation framework, serving primarily as a hypothesis-generating tool. Mandibular V60Gy and oral cavity tumor location were selected as final predictors; however, oral cavity tumor location alone performed nearly as well as the full two-predictor models, suggesting that much of the predictive information may be captured by this predictor. Data augmentation was associated with higher internally cross-validated sensitivity and F1 score estimates than class-weighted baseline training strategies, suggesting improved handling of class imbalance within this cohort rather than demonstrated improvement in generalizable predictive performance. However, given the small number of ORN events, larger multicenter studies with external validation are required before clinical application.

## Data Availability

The analysis code is publicly available on Zenodo (https://zenodo.org/records/22906773), and the study protocol is available on OSF (https://doi.org/10.17605/OSF.IO/XR4SF). More specific patient data are provided in the **Supplementary Material**. The remaining patient-level data cannot be made publicly available due to institutional ethical restrictions and patient privacy, further inquiries can be directed to the corresponding author.
