# Supplementary material for: Machine learning to predict osteoradionecrosis in head and neck cancer
Source: Front Oncol. 2026 Oct 21;16:1955800. doi: 10.3389/fonc.2026.1955800 (PMC13732638; doi:10.3389/fonc.2026.1955800)
Supplement: Supplementary file 1 [file DataSheet1.docx]

**Supplementary Table S1.** Transparent Reporting of a multivariable prediction model for Individual Prognosis Or Diagnosis (TRIPOD+AI) checklist.

| **Section/Topic Item Development Checklist item**  **/ evaluation**^1^ | | | | **Reported on section** |
| --- | --- | --- | --- | --- |
| **TITLE** | | | |  |
| *Title* | 1 | D;E | Identify the study as developing or evaluating the performance of a multivariable prediction model, the target population, and the outcome to be predicted | Title |
| **ABSTRACT** | | | | |
| *Abstract* | 2 | D;E | See TRIPOD+AI for Abstracts checklist | Abstract |
| **INTRODUCTION** | | | | |
| *Background* | 3a | D;E | Explain the healthcare context (including whether diagnostic or prognostic) and rationale for developing or evaluating the prediction model, including references to existing models | Introduction |
|  | 3b | D;E | Describe the target population and the intended purpose of the prediction model in the context of the care pathway, including its intended users (e.g., healthcare professionals, patients, public) | Introduction |
|  | 3c | D;E | Describe any known health inequalities between sociodemographic groups | Introduction |
| *Objectives* | 4 | D;E | Specify the study objectives, including whether the study describes the development or validation of a prediction model (or both) | Introduction |
| **METHODS** | | | | |
| *Data* | 5a | D;E | Describe the sources of data separately for the development and evaluation datasets (e.g., randomised trial, cohort, routine care or registry data), the rationale for using these data, and representativeness of the data | Methods |
|  | 5b | D;E | Specify the dates of the collected participant data, including start and end of participant accrual; and, if applicable, end of follow-up | Methods |
| *Participants* | 6a | D;E | Specify key elements of the study setting (e.g., primary care, secondary care, general population) including the number and location of centres | Methods |
|  | 6b | D;E | Describe the eligibility criteria for study participants | Methods |
|  | 6c | D;E | Give details of any treatments received, and how they were handled during model development or evaluation, if relevant | Methods |
| *Data preparation* | 7 | D;E | Describe any data pre-processing and quality checking, including whether this was similar across relevant sociodemographic groups | Methods |
| *Outcome* | 8a | D;E | Clearly define the outcome that is being predicted and the time horizon, including how and when assessed, the rationale for choosing this outcome, and whether the method of outcome assessment is consistent across sociodemographic groups | Methods |
|  | 8b | D;E | If outcome assessment requires subjective interpretation, describe the qualifications and demographic characteristics of the outcome assessors | Methods |
|  | 8c | D;E | Report any actions to blind assessment of the outcome to be predicted | Methods |
| *Predictors* | 9a | D | Describe the choice of initial predictors (e.g., literature, previous models, all available predictors) and  any pre-selection of predictors before model building | Methods |
|  | 9b | D;E | Clearly define all predictors, including how and when they were measured (and any actions to blind assessment of predictors for the outcome and other predictors) | Methods |
|  | 9c | D;E | If predictor measurement requires subjective interpretation, describe the qualifications and demographic characteristics of the predictor assessors | NA |
| *Sample size* | 10 | D;E | Explain how the study size was arrived at (separately for development and evaluation), and justify that  the study size was sufficient to answer the research question. Include details of any sample size calculation | Methods |
| *Missing data* | 11 | D;E | Describe how missing data were handled. Provide reasons for omitting any data | Methods |
| *Analytical methods* | 12a | D | Describe how the data were used (e.g., for development and evaluation of model performance) in the analysis, including whether the data were partitioned, considering any sample size requirements | Methods |
|  | 12b | D | Depending on the type of model, describe how predictors were handled in the analyses (functional form,  rescaling, transformation, or any standardisation). | Methods |
|  | 12c | D | Specify the type of model, rationale^2^, all model-building steps, including any hyperparameter tuning,  and method for internal validation | Methods |
|  | 12d | D;E | Describe if and how any heterogeneity in estimates of model parameter values and model performance was handled and quantified across clusters (e.g., hospitals, countries). See TRIPOD-Cluster for additional considerations^3^ | NA |
|  | 12e | D;E | Specify all measures and plots used (and their rationale) to evaluate model performance (e.g., discrimination, calibration, clinical utility) and, if relevant, to compare multiple models | Methods |
|  | 12f | E | Describe any model updating (e.g., recalibration) arising from the model evaluation, either overall or for particular sociodemographic groups or settings | Methods |
|  | 12g | E | For model evaluation, describe how the model predictions were calculated (e.g., formula, code, object, application programming interface) | Methods |
| *Class imbalance* | 13 | D;E | If class imbalance methods were used, state why and how this was done, and any subsequent methods to  recalibrate the model or the model predictions | Methods |
| *Fairness* | 14 | D;E | Describe any approaches that were used to address model fairness and their rationale | Methods |
| *Model output* | 15 | D | Specify the output of the prediction model (e.g., probabilities, classification). Provide details and rationale for any classification and how the thresholds were identified | Methods |
| *Training versus*  *evaluation* | 16 | D;E | Identify any differences between the development and evaluation data in healthcare setting, eligibility criteria, outcome, and predictors | Methods |
| *Ethical approval* | 17 | D;E | Name the institutional research board or ethics committee that approved the study and describe the participant-informed consent or the ethics committee waiver of informed consent | Methods |
| **OPEN SCIENCE** | | | | |
| *Funding* | 18a | D;E | Give the source of funding and the role of the funders for the present study | Funding |
| *Conflicts of interest* | 18b | D;E | Declare any conflicts of interest and financial disclosures for all authors | Conflicts of interest |
| *Protocol* | 18c | D;E | Indicate where the study protocol can be accessed or state that a protocol was not prepared | Methods |
| *Registration* | 18d | D;E | Provide registration information for the study, including register name and registration number, or state that the study was not registered | Methods |
| *Data sharing* | 18e | D;E | Provide details of the availability of the study data | Data Availability Statement |
| *Code sharing* | 18f | D;E | Provide details of the availability of the analytical code^4^ | Code Availability Statement |
| **PATIENT & PUBLIC INVOLVEMENT** | | | | |
| *Patient & Public Involvement* | 19 | D;E | Provide details of any patient and public involvement during the design, conduct, reporting, interpretation, or dissemination of the study or state no involvement. | Patient and public involvement |
| **RESULTS** | | | | |
| *Participants* | 20a | D;E | Describe the flow of participants through the study, including the number of participants with and without the outcome and, if applicable, a summary of the follow-up time. A diagram may be helpful. | Results |
|  | 20b | D;E | Report the characteristics overall and, where applicable, for each data source or setting, including the key dates, key predictors (including demographics), treatments received, sample size, number of outcome events, follow-up time, and amount of missing data. A table may be helpful. Report any differences across key demographic groups. | Results |
|  | 20c | E | For model evaluation, show a comparison with the development data of the distribution of important predictors (demographics, predictors, and outcome). | Results |
| *Model development* | 21 | D;E | Specify the number of participants and outcome events in each analysis (e.g., for model development, hyperparameter tuning, model evaluation) | Results |
| *Model specification* | 22 | D | Provide details of the full prediction model (e.g., formula, code, object, application programming interface) to allow predictions in new individuals and to enable third-party evaluation and implementation, including any restrictions to access or re-use (e.g., freely available, proprietary)^5^ | Code Availability Statement |
| *Model performance* | 23a | D;E | Report model performance estimates with confidence intervals, including for any key subgroups (e.g., sociodemographic). Consider plots to aid presentation. | Results |
|  | 23b | D;E | If examined, report results of any heterogeneity in model performance across clusters. See TRIPOD  Cluster for additional details^3^. | NA |
| *Model updating* | 24 | E | Report the results from any model updating, including the updated model and subsequent performance | NA |
| **DISCUSSION** | | | | |
| *Interpretation* | 25 | D;E | Give an overall interpretation of the main results, including issues of fairness in the context of the objectives and previous studies | Discussion |
| *Limitations* | 26 | D;E | Discuss any limitations of the study (such as a non-representative sample, sample size, overfitting, missing data) and their effects on any biases, statistical uncertainty, and generalizability | Discussion |
| *Usability of the model in the context of current care* | 27a | D | Describe how poor quality or unavailable input data (e.g., predictor values) should be assessed and handled when implementing the prediction model | Discussion |
|  | 27b | D | Specify whether users will be required to interact in the handling of the input data or use of the model, and what level of expertise is required of users | Discussion |
|  | 27c | D;E | Discuss any next steps for future research, with a specific view to applicability and generalizability of the model | Discussion |

From: Collins GS, Moons KGM, Dhiman P, et al. BMJ 2024;385:e078378. doi:10.1136/bmj-2023-078378

Abbreviations: NA – Not applicable.
^1^ D=items relevant only to the development of a prediction model; E=items relating solely to the evaluation of a prediction model; D;E=items applicable to both the development and evaluation of a prediction model

^2^ Separately for all model building approaches.

^3^ TRIPOD-Cluster is a checklist of reporting recommendations for studies developing or validating models that explicitly account for clustering or explore heterogeneity in model performance (eg, at different hospitals or centres). Debray et al, BMJ 2023; 380: e071018 [DOI: 10.1136/bmj-2022-071018]

**Supplementary Table S2.** Model configurations and hyperparameters used in the analysis**.**

| **Algorithm** | **Hyperparameter / configuration** | **Value** |
| --- | --- | --- |
| Logistic Regression | Scaling | StandardScaler |
|  | C | 1.0 |
|  | Penalty | L2 |
|  | Solver | lbfgs |
|  | Class weight | balanced |
|  | Maximum iterations | 100 |
|  | Tolerance | 0.0001 |
| Support Vector Machine (SVM) | Scaling | StandardScaler |
|  | Kernel | linear |
|  | C | 1.0 |
|  | Class weight | balanced |
|  | Probability estimates | True |
|  | Tolerance | 0.001 |
| Random Forest | Number of estimators | 100 |
|  | Criterion | gini |
|  | Maximum depth | None |
|  | Maximum features | sqrt |
|  | Minimum samples split | 2 |
|  | Minimum samples leaf | 1 |
|  | Bootstrap | True |
|  | Class weight | balanced |
|  | Random state | 42 |
| XGBoost | Objective | binary:logistic |
|  | Evaluation metric | logloss |
|  | Scale positive weight | Calculated from the class distribution of the training data* |
|  | Random state | 42 |
|  | Other parameters | Library defaults |
| CatBoost | Automatic class weights | Balanced |
|  | Random state | 42 |
|  | Other parameters | Library defaults |
| TabPFN | Number of estimators | 8 |

**Supplementary Table S3.** Software environment and package versions used in the analysis.

| **Software/package** | **Version** |
| --- | --- |
| Python | 3.13.9 |
| NumPy | 2.3.5 |
| pandas | 2.3.3 |
| SciPy | 1.16.3 |
| scikit-learn | 1.7.2 |
| imbalanced-learn | 0.14.0 |
| CatBoost | 1.2.10 |
| XGBoost | 3.2.0 |
| TabPFN | 6.4.1 |
| Matplotlib | 3.10.6 |
| seaborn | 0.13.2 |
| huggingface-hub | 1.5.0 |
| openpyxl | 3.1.5 |

**Supplementary Table S4.** Final logistic regression model specifications for individual ORN risk prediction.

| **Model** | **Term** | **Coefficient** |
| --- | --- | --- |
| Class-weighted baseline LR | Intercept | −2.2601 |
| Class-weighted baseline LR | V60Gy | 0.0311 |
| Class-weighted baseline LR | Oral cavity tumour location | 1.8230 |
| SMOTE-NC augmented LR | Intercept | −3.9324 |
| SMOTE-NC augmented LR | V60Gy | 0.0251 |
| SMOTE-NC augmented LR | Oral cavity tumour location | 4.1168 |

**Supplementary Table S5.** Descriptive and mathematical definitions of metrics.

| **Metric** | **Descriptive Definition** | **Mathematical Definition** |
| --- | --- | --- |
| **Accuracy** | Proportion of all predictions that are correct | Accuracy = (TP + TN) / (TP + TN + FP + FN) |
| **Area Under the ROC Curve (AUC)** | Measures the ability of a model to discriminate between positive and negative classes across all thresholds; equivalent to the probability that a positive instance is ranked higher than a negative one | AUC = ∫₀¹ TPR(FPR⁻¹(u)) du |
| **Brier Score** | Mean squared difference between predicted probabilities and actual outcomes; reflects calibration and accuracy | Brier = (1/N) × Σ (pᵢ − yᵢ)² |
| **F1 Score** | Harmonic mean of precision and sensitivity, balancing false positives and false negatives | F1 = 2 × (Precision × sensitivity) / (Precision + sensitivity) |
| **Log Loss (Cross-Entropy Loss)** | Penalizes incorrect probabilistic predictions, especially those made with high confidence | LogLoss = −(1/N) × Σ [yᵢ log(pᵢ) + (1 − yᵢ) log(1 − pᵢ)] |
| **Precision (Positive Predictive Value)** | Proportion of predicted positive cases that are actually positive | Precision = TP / (TP + FP) |
| **Sensitivity (Recall, True Positive Rate)** | Proportion of actual positive cases correctly identified | sensitivity = TP / (TP + FN) |
| **Specificity (True Negative Rate)** | Proportion of actual negative cases correctly identified | Specificity = TN / (TN + FP) |

**Abbreviations:** acc(Bₘ) – empirical accuracy in bin m; AUC – area under the receiver operating characteristic curve; Bₘ – set of samples in bin m; conf(Bₘ) – mean predicted probability in bin m; F1 – harmonic mean of precision and sensitivity; FN – false negatives; FP – false positives; FPR – false positive rate (FPR = FP / (FP + TN)); LogLoss – logarithmic loss (cross-entropy loss); N – total number of samples; NPV – negative predictive value; pᵢ – predicted probability of the positive class for sample i (pᵢ ∈ [0,1]); PPV – positive predictive value (precision); ROC – receiver operating characteristic; s(x) – model score for input x; TN – true negatives; TP – true positives; TPR – true positive rate (sensitivity) (TPR = TP / (TP + FN)); x⁺, x⁻ – randomly selected positive and negative samples, respectively; yᵢ – true label of sample i (yᵢ ∈ {0,1}); |Bₘ| – number of samples in bin m.

**Supplementary Table S6**. Demographic characteristics of the study population (n=97).

| **Variable** | **Category** | **n** | **%** |
| --- | --- | --- | --- |
| **Sex/gender** | Male | 83 | 85.57 |
|  | Female | 14 | 14.43 |
| **Mean age (years)** | — | 62.6 (SD: 13.83) | — |
| **Marital status** | Married or stable union | 39 | 40.21% |
|  | Single | 23 | 23.71% |
|  | Widowed | 6 | 6.19% |
|  | Previously married (divorced/separated) | 4 | 4.12% |
|  | NA | 25 | 25.77% |
| **Ethnicity** | Brown / Mixed | 77 | 79.38% |
|  | White | 9 | 9.28% |
|  | Black | 7 | 7.22% |
|  | Other (Asian or Indigenous) | 1 | 1.03% |
|  | NA | 3 | 3.09% |
| **Education level** | No formal education | 8 | 8.25% |
|  | Incomplete primary education | 10 | 10.31% |
|  | Complete primary education | 7 | 7.22% |
|  | Complete secondary education | 8 | 8.25% |
|  | Incomplete higher education | 1 | 1.03% |
|  | NA | 63 | 64.95% |

**Abbreviations:** N – Number; NA – Data not available; SD – Standard Deviation; % – percentage.

**Supplementary Table S7.** Clinical and lifestyle characteristics of the study population (n=97).

| **Variable** | **Category** | **n** | **%** |
| --- | --- | --- | --- |
| **Smoking (mean duration in years)** | — | 34.64 | — |
| **Cigarettes per day (mean)** | — | 16.76 | — |
| **Smoking Status** | Current smoker | 29 | 29.90% |
|  | Former smoker | 48 | 49.48% |
|  | Never smoker | 9 | 9.28% |
|  | NA | 11 | 11.34% |
| **Alcohol Status** | Current | 20 | 20.62% |
|  | Former | 46 | 47.42% |
|  | Never | 17 | 17.53% |
|  | NA | 14 | 14.43% |
| **Mean duration of alcohol use (years)** | — | 27 | — |
| **Systemic conditions (yes or no)** | Yes | 55 | 56.70% |
|  | No | 42 | 43.30% |
| **Systemic conditions** | Hypertension | 38 | 39.18% |
|  | Diabetes | 9 | 9.28% |
|  | Pulmonary disease | 7 | 7.22% |
|  | Cancer history | 5 | 5.15% |
|  | Other comorbidities | 25 | 25.77% |
| **Medication use** | Yes | 72 | 74.23 |
|  | No | 25 | 25.77 |
| **Most commonly used medications** | Dipyrone | 21 | 21.65% |
|  | Losartan | 19 | 19.59% |
|  | Morphine | 11 | 11.34% |
|  | Aspirin | 10 | 10.31% |

**Abbreviations**: N – Number; NA – Data not available; % – percentage.

**Supplementary Table S8.** Tumour and treatment characteristics of the study population (n=97).

| **Variable** | **Category** | **n** | **%** |
| --- | --- | --- | --- |
| **Primary tumour location** | Larynx | 34 | 35.05 |
|  | Oral Cavity | 21 | 21.65 |
|  | Oropharynx | 23 | 23.71 |
|  | Nasopharynx | 6 | 6.19 |
|  | Salivary Glands | 6 | 6.19 |
|  | Unspecified primary site | 2 | 2.06 |
|  | Thyroid | 2 | 2.06 |
|  | Hypopharynx | 1 | 1.03 |
|  | Nasal Cavity and Paranasal Sinuses | 1 | 1.03 |
|  | Ear | 1 | 1.03 |
| **Involved regional lymph nodes** | Yes | 38 | 39.18 |
|  | No | 32 | 32.99 |
|  | NA | 27 | 27.84 |
| **Cancer stage (UICC classification)** | I | 13 | 13.4 |
|  | II | 9 | 9.28 |
|  | III | 38 | 39.18 |
|  | IV | 30 | 30.93 |
|  | NA | 7 | 7.22 |
| **Histopathological diagnosis** | Squamous cell carcinoma (SCC) | 88 | 90.72% |
|  | Adenoid cystic carcinoma | 3 | 3.09% |
|  | Mucoepidermoid carcinoma | 1 | 1.03% |
|  | Adenocarcinoma | 1 | 1.03% |
|  | Papillary carcinoma | 1 | 1.03% |
|  | Spindle cell malignant neoplasm | 1 | 1.03% |
|  | Chondrosarcoma | 1 | 1.03% |
| **Histological Grade** | Grade 1 (well differentiated) | 22 | 22.68% |
|  | Grade 2 (moderately differentiated) | 31 | 31.96% |
|  | Grade 3 (poorly differentiated) | 12 | 12.37% |
|  | Grade 4 (undifferentiated/anaplastic) | 1 | 1.03% |
|  | NA | 31 | 31.96% |
| **Status T (TNM)** | T1 | 14 | 14.43% |
|  | T2 | 13 | 13.40% |
|  | T3 | 29 | 29.90% |
|  | T4 | 31 | 31.96% |
|  | NA | 10 | 10.31% |
| **Status N (TNM)** | N0 | 41 | 42.27% |
|  | N1 | 7 | 7.22% |
|  | N2 | 34 | 35.05% |
|  | N3 | 8 | 8.25% |
|  | NA | 7 | 7.22% |
| **Status M (TNM)** | M0 | 68 | 70.10% |
|  | M1 | 4 | 3.88% |
|  | NA | 25 | 25.77% |
| **Concomitant chemotherapy** | Yes | 65 | 67.01% |
|  | No | 32 | 32.99% |
| **Surgery before radiotherapy** | No | 77 | 79.38% |
|  | Yes | 20 | 20.62% |
| **Radiotherapy technique** | 3D-CRT | 97 | 100% |
| **Prescribed total radiation dose (Gy)** | Mean | 64.32 | — |
|  | Minimum | 10 | — |
|  | Maximum | 70 | — |
| **Fractionated dose (Gy)** | 2 Gy | 81 | 83.51% |
|  | 2.25 Gy | 10 | 10.31% |
|  | 2.67 Gy | 1 | 1.03% |
|  | 3 Gy | 2 | 2.06% |
|  | 5 Gy | 2 | 2.06% |
|  | NA | 1 | 1.03% |
| **Purpose of radiotherapy** | Definitive / Radical / Curative | 66 | 68.04% |
|  | Adjuvant | 20 | 20.62% |
|  | Palliative | 5 | 5.15% |
|  | NA | 6 | 6.19% |

**Abbreviations:** Gy – gray; M – distant metastasis stage; N – number; NA – not available; N stage – regional lymph node stage; T – primary tumour stage; % – percentage; 3D-CRT – three-dimensional conformal radiotherapy.

**Supplementary Table S9.** Oral health interventions and complications before and during radiotherapy (n = 97).

| **Intervention/Condition** | **n (yes)** | **%** |
| --- | --- | --- |
| Laser during RT | 80 | 82.47% |
| Tooth Extraction before RT | 25 | 25.77% |
| Laser before RT | 8 | 8.25% |
| Scaling before RT | 7 | 7.22% |
| Endodontic treatment before RT | 6 | 6.19% |
| Scaling during RT | 3 | 3.09% |
| Periodontitis during RT | 2 | 2.06% |

**Abbreviations:** N – Number; RT – Radiotherapy; % – percentage.

**Supplementary Table S10.** Mandible dose–volume histogram means values (n=97).

|  | **Mean** | **Standard deviation (SD)** |
| --- | --- | --- |
| **Total Mandibular Volume (cm³)** | 62.83402 | 14.82002 |
| **Minimum dose (Gy)** | 4.805399 | 6.211521 |
| **Maximum dose (Gy)** | 59.77648 | 23.68971 |
| **Mean dose (Gy)** | 37.33187 | 21.62132 |

**Abbreviations**: cm³ – cubic centimetres; Gy – gray; SD – standard deviation.

**Supplementary Table S11.** Stepwise predictor screening and selection process for the ORN prediction models.

| **Selection stage** | **n** | **Variables** |
| --- | --- | --- |
| **Initial candidate predictors** | **80** | Age; sex; ethnicity; hypertension; diabetes mellitus; alendronate use; prednisone use; endodontic treatment before radiotherapy; dental scaling before radiotherapy; radiotherapy intent; number of radiotherapy fractions; marital status; level of education; histological grade; M status; smoking pack-years; number of involved regional lymph nodes; larynx tumour location; oral cavity tumour location; oropharynx tumour location; nasopharynx tumour location; hypopharynx tumour location; nasal cavity and paranasal sinus tumour location; salivary gland tumour location; thyroid tumour location; unspecified primary site tumour location; ear tumour location; UICC stage III; histopathological diagnosis; pleomorphic adenoma; T status; N status; smoking status; alcohol status; chemotherapy; surgery before radiotherapy; tumour-site radiation dose; fractionated radiation dose; tooth extraction before radiotherapy; mandibular volume; mandibular minimum dose; mandibular maximum dose; mandibular mean dose; D2%; D5%; D10%; D15%; D20%; D25%; D30%; D35%; D40%; D45%; D50%; D55%; D60%; D65%; D70%; D75%; D80%; D85%; D90%; D95%; D97%; D98%; D99%; V5Gy; V10Gy; V15Gy; V20Gy; V25Gy; V30Gy; V35Gy; V40Gy; V45Gy; V50Gy; V55Gy; V60Gy; V65Gy; V70Gy |
| **Excluded based on clinical relevance** | **11 excluded** | Ethnicity; hypertension; diabetes mellitus; alendronate use; prednisone use; endodontic treatment before radiotherapy; dental scaling before radiotherapy; radiotherapy intent; number of radiotherapy fractions; marital status; level of education |
| **Excluded due to >20% missing data** | **4 excluded** | Histological grade; M status; smoking pack-years; number of involved regional lymph nodes |
| **Entered multicollinearity screening** | **65** | Age; sex; larynx tumour location; oral cavity tumour location; oropharynx tumour location; nasopharynx tumour location; hypopharynx tumour location; nasal cavity and paranasal sinus tumour location; salivary gland tumour location; thyroid tumour location; unknown tumour location; ear tumour location; UICC stage III; histopathological diagnosis; pleomorphic adenoma; T status; N status; smoking status; alcohol status; chemotherapy; surgery before radiotherapy; tumour-site radiation dose; fractionated radiation dose; tooth extraction before radiotherapy; mandibular volume; mandibular minimum dose; mandibular maximum dose; mandibular mean dose; D2%; D5%; D10%; D15%; D20%; D25%; D30%; D35%; D40%; D45%; D50%; D55%; D60%; D65%; D70%; D75%; D80%; D85%; D90%; D95%; D97%; D98%; D99%; V5Gy; V10Gy; V15Gy; V20Gy; V25Gy; V30Gy; V35Gy; V40Gy; V45Gy; V50Gy; V55Gy; V60Gy; V65Gy; V70Gy |
| **Final predictors after forward stepwise selection** | **2** | **Mandibular V60Gy; oral cavity tumour location** |

**Abbreviations:** ORN, osteoradionecrosis; UICC, Union for International Cancer Control; D*x*%, dose received by *x*% of the mandibular volume; V*x*Gy, percentage of the mandibular volume receiving at least *x* Gy; Gy, gray.

**Supplementary Table S12.** Selection frequencies of the six most frequently selected predictors across 5,000 iterations.

| **Feature** | **Times selected** | **Selection frequency (%)** | **Training folds retained after correlation screening** | **Training folds removed during correlation screening** | **Correlation-screening retention (%)** |
| --- | --- | --- | --- | --- | --- |
| Oral cavity tumour location | 2,174 | 43.48 | 50 | 0 | 100 |
| Mandibular V60Gy | 1,951 | 39.02 | 40 | 10 | 80 |
| Histopathological diagnosis | 1,102 | 22.04 | 50 | 0 | 100 |
| Alcohol status | 857 | 17.14 | 50 | 0 | 100 |
| Cancer surgery before RT | 643 | 12.86 | 50 | 0 | 100 |
| Mandibular V70Gy | 621 | 12.42 | 12 | 38 | 24 |

**Note:** Selection frequency represents the proportion of the 5,000 bootstrap selection iterations in which each feature was retained. Correlation-screening retention represents the number and percentage of the 50 training folds in which each feature remained available after multicollinearity screening. Features removed during correlation screening were not eligible for forward stepwise selection within the corresponding training fold. **Abbreviations:** Gy, gray; RT, radiotherapy; V60Gy, percentage of the mandibular volume receiving at least 60 Gy; V70Gy, percentage of the mandibular volume receiving at least 70 Gy.

**Supplementary Table S13.** Comprehensive performance metrics for all ML algorithms across different class-imbalance strategies (Mean and 95% CI).

| **Approach** | **Model** | **Threshold** | **AUC** | **Sensitivity** | **Specificity** | **Accuracy** | **Precision** | **F1 Score** | **Brier** | **Logloss** |
| --- | --- | --- | --- | --- | --- | --- | --- | --- | --- | --- |
| **Class-weighted baseline** | CatBoost | 0.50 | 0.82 (0.66–0.97) | 0.54 (0.04–1.00) | 0.86 (0.75–0.97) | 0.84 (0.75–0.92) | 0.29 (0.01–0.64) | 0.33 (0.02–0.65) | 0.13 (0.06–0.19) | 0.40 (0.23–0.57) |
| **Class-weighted baseline** | Logistic Regression | 0.50 | 0.87 (0.74–0.99) | 0.74 (0.35–1.00) | 0.83 (0.72–0.95) | 0.82 (0.72–0.93) | 0.33 (0.07–0.64) | 0.42 (0.13–0.71) | 0.15 (0.09–0.21) | 0.48 (0.33–0.63) |
| **Class-weighted baseline** | Random Forest | 0.50 | 0.73 (0.55–0.90) | 0.09 (0.00–0.23) | 0.96 (0.92–1.00) | 0.89 (0.85–0.93) | 0.15 (0.00–0.42) | 0.08 (0.00–0.17) | 0.09 (0.06–0.12) | 0.87 (0.01–1.73) |
| **Class-weighted baseline** | SVM | 0.50 | 0.85 (0.69–0.99) | 0.00 (NE) | 0.999 (0.998–1.000) | 0.92 (0.89–0.94) | **NA** | 0.00 (NE) | 0.07 (0.05–0.09) | 0.26 (0.18–0.34) |
| **Baseline** | TabPFN | 0.50 | 0.85 (0.72–0.97) | 0.03 (0.00–0.09) | 0.98 (0.96–1.00) | 0.90 (0.86–0.94) | 0.12 (0.00–0.55) | 0.02 (0.00–0.05) | 0.07 (0.05–0.10) | 0.25 (0.16–0.35) |
| **Class-weighted baseline** | XGBoost | 0.50 | 0.78 (0.65–0.91) | 0.04 (0.00–0.08) | 0.94 (0.87–1.00) | 0.87 (0.80–0.93) | 0.04 (0.00–0.09) | 0.03 (0.00–0.06) | 0.12 (0.06–0.17) | 0.42 (0.21–0.62) |
| **Threshold adjust** | CatBoost | 0.66 | 0.82 (0.66–0.97) | 0.41 (0.00–0.95) | 0.90 (0.80–0.99) | 0.86 (0.77–0.94) | 0.28 (0.00–0.63) | 0.27 (0.00–0.59) | 0.13 (0.06–0.19) | 0.40 (0.23–0.57) |
| **Threshold adjust** | Logistic Regression | 0.67 | 0.87 (0.74–0.99) | 0.63 (0.12–1.00) | 0.87 (0.77–0.97) | 0.85 (0.76–0.94) | 0.34 (0.03–0.71) | 0.39 (0.05–0.73) | 0.15 (0.09–0.21) | 0.48 (0.33–0.63) |
| **Threshold adjust** | Random Forest | 0.56 | 0.73 (0.55–0.90) | 0.09 (0.00–0.23) | 0.97 (0.93–1.00) | 0.90 (0.85–0.94) | 0.16 (0.00–0.47) | 0.08 (0.00–0.17) | 0.09 (0.06–0.12) | 0.87 (0.01–1.73) |
| **Threshold adjust** | SVM | 0.13 | 0.85 (0.69–0.99) | 0.65 (0.15–1.00) | 0.85 (0.75–0.95) | 0.83 (0.75–0.92) | 0.30 (0.04–0.60) | 0.38 (0.07–0.68) | 0.07 (0.05–0.09) | 0.26 (0.18–0.33) |
| **Threshold adjust** | TabPFN | 0.16 | 0.85 (0.72–0.97) | 0.66 (0.10–1.00) | 0.85 (0.73–0.97) | 0.83 (0.74–0.93) | 0.31 (0.03–0.62) | 0.37 (0.03–0.72) | 0.07 (0.05–0.10) | 0.25 (0.16–0.35) |
| **Threshold adjust** | XGBoost | 0.91 | 0.78 (0.65–0.91) | 0.04 (0.00–0.08) | 0.95 (0.89–1.00) | 0.88 (0.82–0.93) | 0.06 (0.00–0.13) | 0.03 (0.00–0.07) | 0.12 (0.06–0.17) | 0.42 (0.21–0.62) |
| **Data Aug** | CatBoost | 0.50 | 0.84 (0.67–0.99) | 0.77 (0.40–1.00) | 0.86 (0.76–0.96) | 0.85 (0.76–0.95) | 0.38 (0.09–0.73) | 0.47 (0.16–0.79) | 0.12 (0.05–0.20) | 0.42 (0.19–0.65) |
| **Data Aug** | Logistic Regression | 0.50 | 0.87 (0.73–0.99) | 0.77 (0.40–1.00) | 0.84 (0.74–0.94) | 0.84 (0.75–0.92) | 0.33 (0.09–0.61) | 0.44 (0.16–0.71) | 0.13 (0.06–0.20) | 0.40 (0.21–0.59) |
| **Data Aug** | Random Forest | 0.50 | 0.84 (0.67–0.99) | 0.73 (0.27–1.00) | 0.86 (0.77–0.95) | 0.85 (0.77–0.93) | 0.34 (0.07–0.64) | 0.43 (0.11–0.74) | 0.13 (0.06–0.21) | 2.12 (0.00–4.28) |
| **Data Aug** | SVM | 0.50 | 0.84 (0.68–0.99) | 0.77 (0.40–1.00) | 0.83 (0.74–0.93) | 0.82 (0.74–0.91) | 0.31 (0.10–0.54) | 0.41 (0.15–0.68) | 0.13 (0.07–0.20) | 0.41 (0.24–0.59) |
| **Data Aug** | TabPFN | 0.50 | 0.85 (0.71–0.98) | 0.77 (0.40–1.00) | 0.86 (0.76–0.96) | 0.85 (0.76–0.95) | 0.38 (0.09–0.73) | 0.48 (0.16–0.80) | 0.13 (0.05–0.20) | 0.42 (0.20–0.64) |
| **Data Aug** | XGBoost | 0.50 | 0.82 (0.63–0.99) | 0.72 (0.24–1.00) | 0.86 (0.75–0.97) | 0.84 (0.74–0.94) | 0.35 (0.05–0.72) | 0.43 (0.08–0.79) | 0.14 (0.05–0.22) | 0.52 (0.22–0.81) |

**Abbreviations:** AUC – Area Under the Receiver Operating Characteristic Curve; Brier – Brier Score; CatBoost – Categorical Boosting; CI – Confidence Interval; LR – Logistic Regression; NA – Not Available; NE – Not Estimable; RF – Random Forest; SVM – Support Vector Machine; TabPFN – Tabular Prior-Data Fitted Network; XGBoost – Extreme Gradient Boosting.

**Supplementary Table S14.** Statistical Significance Matrix of F1 Scores (Nadeau–Bengio Corrected Paired t-Test with Holm Adjustment). Red cells indicate statistically significant pairwise differences (Holm-adjusted p < 0.05), while blue cells indicate no statistically significant difference.

| **Approach / Model** | **Aug-CB** | **Aug-LR** | **Aug-RF** | **Aug-SVM** | **Aug-PFN** | **Aug-XGB** | **Base-CB** | **Base-LR** | **Base-RF** | **Base-SVM** | **Base-PFN** | **Base-XGB** | **Thres-CB** | **Thres-LR** | **Thres-RF** | **Thres-SVM** | **Thres-PFN** | **Thres-XGB** |
| --- | --- | --- | --- | --- | --- | --- | --- | --- | --- | --- | --- | --- | --- | --- | --- | --- | --- | --- |
| **Aug-CB** | 1.00 | 1.00 | 1.00 | 1.00 | 1.00 | 1.00 | 1.00 | 1.00 | 1.00 | **0.05** | 0.31 | 0.35 | 1.00 | 1.00 | 1.00 | 1.00 | 1.00 | 0.44 |
| **Aug-LR** | 1.00 | 1.00 | 1.00 | 1.00 | 1.00 | 1.00 | 1.00 | 1.00 | 1.00 | **0.04** | 0.29 | 0.32 | 1.00 | 1.00 | 1.00 | 1.00 | 1.00 | 0.41 |
| **Aug-RF** | 1.00 | 1.00 | 1.00 | 1.00 | 1.00 | 1.00 | 1.00 | 1.00 | 1.00 | 0.13 | 0.53 | 0.72 | 1.00 | 1.00 | 1.00 | 1.00 | 1.00 | 0.88 |
| **Aug-SVM** | 1.00 | 1.00 | 1.00 | 1.00 | 1.00 | 1.00 | 1.00 | 1.00 | 1.00 | **0.04** | 0.33 | 0.38 | 1.00 | 1.00 | 1.00 | 1.00 | 1.00 | 0.48 |
| **Aug-PFN** | 1.00 | 1.00 | 1.00 | 1.00 | 1.00 | 1.00 | 1.00 | 1.00 | 1.00 | 0.06 | 0.33 | 0.38 | 1.00 | 1.00 | 1.00 | 1.00 | 1.00 | 0.46 |
| **Aug-XGB** | 1.00 | 1.00 | 1.00 | 1.00 | 1.00 | 1.00 | 1.00 | 1.00 | 1.00 | 0.41 | 1.00 | 1.00 | 1.00 | 1.00 | 1.00 | 1.00 | 1.00 | 1.00 |
| **Base-CB** | 1.00 | 1.00 | 1.00 | 1.00 | 1.00 | 1.00 | 1.00 | 1.00 | 1.00 | 1.00 | 1.00 | 1.00 | 1.00 | 1.00 | 1.00 | 1.00 | 1.00 | 1.00 |
| **Base-LR** | 1.00 | 1.00 | 1.00 | 1.00 | 1.00 | 1.00 | 1.00 | 1.00 | 1.00 | 0.09 | 0.51 | 0.63 | 1.00 | 1.00 | 1.00 | 1.00 | 1.00 | 0.79 |
| **Base-RF** | 1.00 | 1.00 | 1.00 | 1.00 | 1.00 | 1.00 | 1.00 | 1.00 | 1.00 | 1.00 | 1.00 | 1.00 | 1.00 | 1.00 | 1.00 | 1.00 | 1.00 | 1.00 |
| **Base-SVM** | **0.05** | **0.04** | 0.13 | **0.04** | 0.06 | 0.41 | 1.00 | 0.09 | 1.00 | 1.00 | 1.00 | 1.00 | 1.00 | 0.63 | 1.00 | 0.44 | 1.00 | 1.00 |
| **Base-PFN** | 0.31 | 0.29 | 0.53 | 0.33 | 0.33 | 1.00 | 1.00 | 0.51 | 1.00 | 1.00 | 1.00 | 1.00 | 1.00 | 1.00 | 1.00 | 1.00 | 1.00 | 1.00 |
| **Base-XGB** | 0.35 | 0.32 | 0.72 | 0.38 | 0.38 | 1.00 | 1.00 | 0.63 | 1.00 | 1.00 | 1.00 | 1.00 | 1.00 | 1.00 | 1.00 | 1.00 | 1.00 | 1.00 |
| **Thres-CB** | 1.00 | 1.00 | 1.00 | 1.00 | 1.00 | 1.00 | 1.00 | 1.00 | 1.00 | 1.00 | 1.00 | 1.00 | 1.00 | 1.00 | 1.00 | 1.00 | 1.00 | 1.00 |
| **Thres-LR** | 1.00 | 1.00 | 1.00 | 1.00 | 1.00 | 1.00 | 1.00 | 1.00 | 1.00 | 0.63 | 1.00 | 1.00 | 1.00 | 1.00 | 1.00 | 1.00 | 1.00 | 1.00 |
| **Thres-RF** | 1.00 | 1.00 | 1.00 | 1.00 | 1.00 | 1.00 | 1.00 | 1.00 | 1.00 | 1.00 | 1.00 | 1.00 | 1.00 | 1.00 | 1.00 | 1.00 | 1.00 | 1.00 |
| **Thres-SVM** | 1.00 | 1.00 | 1.00 | 1.00 | 1.00 | 1.00 | 1.00 | 1.00 | 1.00 | 0.44 | 1.00 | 1.00 | 1.00 | 1.00 | 1.00 | 1.00 | 1.00 | 1.00 |
| **Thres-PFN** | 1.00 | 1.00 | 1.00 | 1.00 | 1.00 | 1.00 | 1.00 | 1.00 | 1.00 | 1.00 | 1.00 | 1.00 | 1.00 | 1.00 | 1.00 | 1.00 | 1.00 | 1.00 |
| **Thres-XGB** | 0.44 | 0.41 | 0.88 | 0.48 | 0.46 | 1.00 | 1.00 | 0.79 | 1.00 | 1.00 | 1.00 | 1.00 | 1.00 | 1.00 | 1.00 | 1.00 | 1.00 | 1.00 |

**Abbreviations:** Aug – data augmentation; CatBoost/CB – categorical boosting; F1 Score – harmonic mean of precision and sensitivity; LR – logistic regression; PFN – tabular prior-data fitted network; RF – random forest; Spec – specificity; SVM – support vector machine; Thres/Threshold – classification threshold; Base – Class-weighted baseline models; XGB/XGBoost – extreme gradient boosting.

**Supplementary Table S15.** Statistical Significance Matrix of Sensitivity Scores (Nadeau–Bengio Corrected Paired t-Test with Holm Adjustment). Red cells indicate statistically significant pairwise differences (Holm-adjusted p < 0.05), while blue cells indicate no statistically significant difference.

| **Approach / Model** | **Aug-CB** | **Aug-LR** | **Aug-RF** | **Aug-SVM** | **Aug-PFN** | **Aug-XGB** | **Base-CB** | **Base-LR** | **Base-RF** | **Base-SVM** | **Base-PFN** | **Base-XGB** | **Thres-CB** | **Thres-LR** | **Thres-RF** | **Thres-SVM** | **Thres-PFN** | **Thres-XGB** |
| --- | --- | --- | --- | --- | --- | --- | --- | --- | --- | --- | --- | --- | --- | --- | --- | --- | --- | --- |
| **Aug-CB** | 1.00 | 1.00 | 1.00 | 1.00 | 1.00 | 1.00 | 1.00 | 1.00 | 0.19 | **0.00** | **0.02** | 0.05 | 1.00 | 1.00 | 0.19 | 1.00 | 1.00 | 0.05 |
| **Aug-LR** | 1.00 | 1.00 | 1.00 | 1.00 | 1.00 | 1.00 | 1.00 | 1.00 | 0.19 | **0.00** | **0.02** | 0.05 | 1.00 | 1.00 | 0.19 | 1.00 | 1.00 | 0.05 |
| **Aug-RF** | 1.00 | 1.00 | 1.00 | 1.00 | 1.00 | 1.00 | 1.00 | 1.00 | 0.54 | 0.05 | 0.11 | 0.22 | 1.00 | 1.00 | 0.54 | 1.00 | 1.00 | 0.22 |
| **Aug-SVM** | 1.00 | 1.00 | 1.00 | 1.00 | 1.00 | 1.00 | 1.00 | 1.00 | 0.19 | **0.00** | **0.02** | 0.05 | 1.00 | 1.00 | 0.19 | 1.00 | 1.00 | 0.05 |
| **Aug-PFN** | 1.00 | 1.00 | 1.00 | 1.00 | 1.00 | 1.00 | 1.00 | 1.00 | 0.19 | **0.00** | **0.02** | 0.05 | 1.00 | 1.00 | 0.19 | 1.00 | 1.00 | 0.05 |
| **Aug-XGB** | 1.00 | 1.00 | 1.00 | 1.00 | 1.00 | 1.00 | 1.00 | 1.00 | 0.70 | 0.09 | 0.17 | 0.31 | 1.00 | 1.00 | 0.70 | 1.00 | 1.00 | 0.31 |
| **Base-CB** | 1.00 | 1.00 | 1.00 | 1.00 | 1.00 | 1.00 | 1.00 | 1.00 | 1.00 | 1.00 | 1.00 | 1.00 | 1.00 | 1.00 | 1.00 | 1.00 | 1.00 | 1.00 |
| **Base-LR** | 1.00 | 1.00 | 1.00 | 1.00 | 1.00 | 1.00 | 1.00 | 1.00 | 0.23 | **0.01** | **0.03** | 0.07 | 1.00 | 1.00 | 0.23 | 1.00 | 1.00 | 0.07 |
| **Base-RF** | 0.19 | 0.19 | 0.54 | 0.19 | 0.19 | 0.70 | 1.00 | 0.23 | 1.00 | 1.00 | 1.00 | 1.00 | 1.00 | 1.00 | 1.00 | 1.00 | 1.00 | 1.00 |
| **Base-SVM** | **0.00** | **0.00** | 0.05 | **0.00** | **0.00** | 0.09 | 1.00 | **0.01** | 1.00 | 1.00 | 1.00 | 1.00 | 1.00 | 0.35 | 1.00 | 0.23 | 0.56 | 1.00 |
| **Base-PFN** | **0.02** | **0.02** | 0.11 | **0.02** | **0.02** | 0.17 | 1.00 | **0.03** | 1.00 | 1.00 | 1.00 | 1.00 | 1.00 | 0.54 | 1.00 | 0.37 | 0.83 | 1.00 |
| **Base-XGB** | 0.05 | 0.05 | 0.22 | 0.05 | 0.05 | 0.31 | 1.00 | 0.07 | 1.00 | 1.00 | 1.00 | 1.00 | 1.00 | 0.85 | 1.00 | 0.78 | 1.00 | 1.00 |
| **Thres-CB** | 1.00 | 1.00 | 1.00 | 1.00 | 1.00 | 1.00 | 1.00 | 1.00 | 1.00 | 1.00 | 1.00 | 1.00 | 1.00 | 1.00 | 1.00 | 1.00 | 1.00 | 1.00 |
| **Thres-LR** | 1.00 | 1.00 | 1.00 | 1.00 | 1.00 | 1.00 | 1.00 | 1.00 | 1.00 | 0.35 | 0.54 | 0.85 | 1.00 | 1.00 | 1.00 | 1.00 | 1.00 | 0.85 |
| **Thres-RF** | 0.19 | 0.19 | 0.54 | 0.19 | 0.19 | 0.70 | 1.00 | 0.23 | 1.00 | 1.00 | 1.00 | 1.00 | 1.00 | 1.00 | 1.00 | 1.00 | 1.00 | 1.00 |
| **Thres-SVM** | 1.00 | 1.00 | 1.00 | 1.00 | 1.00 | 1.00 | 1.00 | 1.00 | 1.00 | 0.23 | 0.37 | 0.78 | 1.00 | 1.00 | 1.00 | 1.00 | 1.00 | 0.78 |
| **Thres-PFN** | 1.00 | 1.00 | 1.00 | 1.00 | 1.00 | 1.00 | 1.00 | 1.00 | 1.00 | 0.56 | 0.83 | 1.00 | 1.00 | 1.00 | 1.00 | 1.00 | 1.00 | 1.00 |
| **Thres-XGB** | 0.05 | 0.05 | 0.22 | 0.05 | 0.05 | 0.31 | 1.00 | 0.07 | 1.00 | 1.00 | 1.00 | 1.00 | 1.00 | 0.85 | 1.00 | 0.78 | 1.00 | 1.00 |

**Abbreviations:** Aug – data augmentation; CatBoost/CB – categorical boosting; F1 Score – harmonic mean of precision and sensitivity; LR – logistic regression; PFN – tabular prior-data fitted network; RF – random forest; Spec – specificity; SVM – support vector machine; Thres/Threshold – classification threshold; Base – baseusted models; XGB/XGBoost – extreme gradient boosting.

**Supplementary Table S16.** Absolute classification counts across repeated out-of-fold predictions.

| **Approach** | **Model** | **Unique ORN cases** | **TP** | **FN** | **FP** | **TN** | **Pooled sensitivity** | **Pooled PPV** |
| --- | --- | --- | --- | --- | --- | --- | --- | --- |
| Class-weighted baseline | Logistic Regression | 8 | 56 | 24 | 148 | 742 | 0.70 | 0.27 |
| Threshold adjustment | Logistic Regression | 8 | 49 | 31 | 116 | 774 | 0.61 | 0.30 |
| Data augmentation | Logistic Regression | 8 | 59 | 21 | 138 | 752 | 0.74 | 0.30 |
| Class-weighted baseline | SVM | 8 | 0 | 80 | 1 | 889 | 0.00 | 0.00 |
| Threshold adjustment | SVM | 8 | 50 | 30 | 132 | 758 | 0.63 | 0.27 |
| Data augmentation | SVM | 8 | 59 | 21 | 150 | 740 | 0.74 | 0.28 |
| Class-weighted baseline | Random Forest | 8 | 8 | 72 | 33 | 857 | 0.10 | 0.20 |
| Threshold adjustment | Random Forest | 8 | 8 | 72 | 30 | 860 | 0.10 | 0.21 |
| Data augmentation | Random Forest | 8 | 55 | 25 | 120 | 770 | 0.69 | 0.31 |
| Class-weighted baseline | XGBoost | 8 | 4 | 76 | 55 | 835 | 0.05 | 0.07 |
| Threshold adjustment | XGBoost | 8 | 4 | 76 | 44 | 846 | 0.05 | 0.08 |
| Data augmentation | XGBoost | 8 | 54 | 26 | 125 | 765 | 0.68 | 0.30 |
| Class-weighted baseline | CatBoost | 8 | 43 | 37 | 121 | 769 | 0.54 | 0.26 |
| Threshold adjustment | CatBoost | 8 | 31 | 49 | 89 | 801 | 0.39 | 0.26 |
| Data augmentation | CatBoost | 8 | 59 | 21 | 120 | 770 | 0.74 | 0.33 |
| Baseline | TabPFN | 8 | 2 | 78 | 16 | 874 | 0.03 | 0.11 |
| Threshold adjustment | TabPFN | 8 | 51 | 29 | 131 | 759 | 0.64 | 0.28 |
| Data augmentation | TabPFN | 8 | 59 | 21 | 120 | 770 | 0.74 | 0.33 |

**Note:** Absolute counts represent repeated out-of-fold predictions and should not be interpreted as counts of independent patients. The cohort contained eight unique ORN cases, each contributing one out-of-fold prediction in each of the 10 cross-validation repetitions, resulting in 80 OOF predictions for ORN cases. TP, true positive; FN, false negative; FP, false positive; TN, true negative; PPV, positive predictive value; OOF, out-of-fold.

**Supplementary Figure S17. Calibration plots of the osteoradionecrosis prediction models across modelling strategies.** Patient-level aggregated out-of-fold (OOF) predicted probabilities were used to construct calibration curves for (A) class-weighted baseline modelling, (B) probability threshold adjustment, and (C) data augmentation. The diagonal line represents ideal calibration, while the model-specific curves represent logistic recalibration based on the aggregated OOF predictions. Shaded areas represent 95% confidence bands obtained by patient-level bootstrap resampling. Abbreviations: OOF, out-of-fold; LR, logistic regression; SVM, support vector machine; RF, random forest.

**Supplementary Table S18.** Calibration intercept and slope of the ORN prediction models.

| **Approach** | **Model** | **Calibration intercept (95% CI)** | **Calibration slope (95% CI)** |
| --- | --- | --- | --- |
| Class-weighted baseline | Logistic Regression | −2.19 (−4.18 to −1.56) | 0.72 (0.12 to 1.69) |
| Class-weighted baseline | SVM | 0.40 (−3.06 to 3.36) | 1.18 (−0.13 to 2.82) |
| Class-weighted baseline | Random Forest | −0.99 (−1.99 to −0.02) | 0.25 (0.15 to 0.50) |
| Class-weighted baseline | XGBoost | −1.49 (−2.77 to −0.33) | 0.31 (0.05 to 0.64) |
| Class-weighted baseline | CatBoost | −1.78 (−3.27 to −0.99) | 0.48 (−0.00 to 1.17) |
| Baseline | TabPFN | −0.48 (−2.56 to 1.17) | 0.75 (0.10 to 1.49) |
| Threshold adjustment | Logistic Regression | −2.19 (−4.18 to −1.56) | 0.72 (0.12 to 1.69) |
| Threshold adjustment | SVM | 0.45 (−2.93 to 3.44) | 1.21 (−0.07 to 2.85) |
| Threshold adjustment | Random Forest | −0.99 (−1.99 to −0.02) | 0.25 (0.15 to 0.50) |
| Threshold adjustment | XGBoost | −1.49 (−2.77 to −0.33) | 0.31 (0.05 to 0.64) |
| Threshold adjustment | CatBoost | −1.78 (−3.27 to −0.99) | 0.48 (−0.00 to 1.17) |
| Threshold adjustment | TabPFN | −0.48 (−2.56 to 1.17) | 0.75 (0.10 to 1.49) |
| Data augmentation | Logistic Regression | −1.84 (−3.86 to −1.14) | 0.52 (0.19 to 1.39) |
| Data augmentation | SVM | −1.86 (−3.62 to −1.19) | 0.52 (0.20 to 1.37) |
| Data augmentation | Random Forest | −1.78 (−2.95 to −1.04) | 0.20 (0.10 to 0.57) |
| Data augmentation | XGBoost | −1.84 (−3.16 to −1.15) | 0.38 (0.09 to 0.78) |
| Data augmentation | CatBoost | −1.72 (−3.19 to −1.01) | 0.43 (0.13 to 0.94) |
| Data augmentation | TabPFN | −1.73 (−3.13 to −1.02) | 0.43 (0.14 to 0.96) |

Calibration intercept and slope were estimated from patient-level aggregated out-of-fold predicted probabilities. The mean of 10 repeated out-of-fold predictions was calculated for each patient. The 95% confidence intervals were obtained using 1,000 patient-level bootstrap resamples. Ideal calibration corresponds to an intercept of 0 and a slope of 1. Estimates should be interpreted cautiously given the small number of ORN events (n = 8).

**Supplementary Table S19**. Model performance in the sensitivity analysis excluding patients with less than one year of follow-up.

| **Approach** | **Model** | **Threshold** | **AUC** | **Sensitivity** | **Specificity** | **Accuracy** | **Precision** | **F1 Score** | **Brier** | **Logloss** |
| --- | --- | --- | --- | --- | --- | --- | --- | --- | --- | --- |
| Class-weighted baseline | CatBoost | 0.50 | 0.78 (0.58–0.99) | 0.50 (0.08–0.92) | 0.86 (0.76–0.95) | 0.82 (0.74–0.90) | 0.30 (0.02–0.58) | 0.34 (0.07–0.61) | 0.14 (0.09–0.19) | 0.45 (0.28–0.61) |
| Class-weighted baseline | Logistic Regression | 0.50 | 0.85 (0.66–1.00) | 0.66 (0.23–1.00) | 0.81 (0.71–0.91) | 0.80 (0.71–0.89) | 0.31 (0.07–0.56) | 0.40 (0.13–0.67) | 0.16 (0.10–0.21) | 0.49 (0.34–0.65) |
| Class-weighted baseline | Random Forest | 0.50 | 0.69 (0.47–0.90) | 0.17 (0.00–0.50) | 0.94 (0.89–1.00) | 0.86 (0.81–0.91) | 0.16 (0.00–0.46) | 0.15 (0.00–0.42) | 0.12 (0.07–0.16) | 1.38 (0.19–2.58) |
| Class-weighted baseline | SVM | 0.50 | 0.82 (0.59–1.00) | 0.00 (NE) | 1.00 (NE) | 0.89 (0.86–0.93) | NA | 0.00 (NE) | 0.09 (0.06–0.11) | 0.31 (0.23–0.38) |
| Baseline | TabPFN | 0.50 | 0.85 (0.67–1.00) | 0.08 (0.00–0.35) | 0.97 (0.91–1.00) | 0.87 (0.82–0.93) | 0.07 (0.00–0.30) | 0.07 (0.00–0.29) | 0.09 (0.06–0.12) | 0.30 (0.18–0.42) |
| Class-weighted baseline | XGBoost | 0.50 | 0.73 (0.57–0.89) | 0.15 (0.00–0.47) | 0.92 (0.85–1.00) | 0.84 (0.78–0.91) | 0.10 (0.00–0.32) | 0.11 (0.00–0.34) | 0.14 (0.09–0.19) | 0.49 (0.29–0.68) |
| Threshold adjustment | CatBoost | 0.63 | 0.78 (0.58–0.99) | 0.38 (0.00–0.81) | 0.89 (0.79–1.00) | 0.84 (0.76–0.92) | 0.26 (0.00–0.58) | 0.27 (0.00–0.57) | 0.14 (0.09–0.19) | 0.45 (0.28–0.61) |
| Threshold adjustment | Logistic Regression | 0.65 | 0.85 (0.66–1.00) | 0.60 (0.17–1.00) | 0.85 (0.75–0.95) | 0.83 (0.74–0.91) | 0.34 (0.05–0.64) | 0.40 (0.11–0.69) | 0.16 (0.10–0.21) | 0.49 (0.34–0.65) |
| Threshold adjustment | Random Forest | 0.60 | 0.69 (0.47–0.90) | 0.16 (0.00–0.48) | 0.95 (0.89–1.00) | 0.87 (0.81–0.92) | 0.17 (0.00–0.51) | 0.15 (0.00–0.42) | 0.12 (0.07–0.16) | 1.38 (0.19–2.58) |
| Threshold adjustment | SVM | 0.19 | 0.82 (0.59–1.00) | 0.54 (0.09–0.99) | 0.84 (0.74–0.94) | 0.81 (0.73–0.89) | 0.29 (0.01–0.57) | 0.34 (0.07–0.61) | 0.09 (0.06–0.12) | 0.31 (0.23–0.40) |
| Threshold adjustment | TabPFN | 0.24 | 0.85 (0.67–1.00) | 0.52 (0.06–0.98) | 0.90 (0.80–0.99) | 0.85 (0.78–0.93) | 0.32 (0.00–0.66) | 0.36 (0.04–0.68) | 0.09 (0.06–0.12) | 0.30 (0.18–0.42) |
| Threshold adjustment | XGBoost | 0.89 | 0.73 (0.57–0.89) | 0.11 (0.00–0.39) | 0.94 (0.88–1.00) | 0.85 (0.79–0.91) | 0.08 (0.00–0.29) | 0.08 (0.00–0.30) | 0.14 (0.09–0.19) | 0.49 (0.29–0.68) |
| Data augmentation | CatBoost | 0.50 | 0.80 (0.55–1.00) | 0.73 (0.32–1.00) | 0.84 (0.75–0.94) | 0.83 (0.74–0.93) | 0.39 (0.10–0.67) | 0.48 (0.18–0.79) | 0.13 (0.06–0.20) | 0.44 (0.19–0.69) |
| Data augmentation | Logistic Regression | 0.50 | 0.85 (0.66–1.00) | 0.71 (0.29–1.00) | 0.83 (0.73–0.92) | 0.82 (0.72–0.91) | 0.34 (0.08–0.59) | 0.44 (0.15–0.73) | 0.13 (0.07–0.20) | 0.42 (0.22–0.61) |
| Data augmentation | Random Forest | 0.50 | 0.80 (0.58–1.00) | 0.69 (0.28–1.00) | 0.87 (0.78–0.96) | 0.85 (0.76–0.94) | 0.43 (0.10–0.75) | 0.50 (0.17–0.82) | 0.13 (0.06–0.21) | 2.33 (0.21–4.46) |
| Data augmentation | SVM | 0.50 | 0.81 (0.57–1.00) | 0.69 (0.26–1.00) | 0.81 (0.72–0.90) | 0.80 (0.72–0.88) | 0.29 (0.09–0.50) | 0.40 (0.14–0.66) | 0.15 (0.08–0.21) | 0.46 (0.26–0.66) |
| Data augmentation | TabPFN | 0.50 | 0.83 (0.65–1.00) | 0.73 (0.32–1.00) | 0.85 (0.76–0.94) | 0.84 (0.75–0.93) | 0.40 (0.11–0.68) | 0.49 (0.19–0.80) | 0.13 (0.06–0.20) | 0.44 (0.19–0.69) |
| Data augmentation | XGBoost | 0.50 | 0.79 (0.56–1.00) | 0.65 (0.22–1.00) | 0.85 (0.75–0.95) | 0.83 (0.73–0.93) | 0.34 (0.08–0.61) | 0.43 (0.14–0.72) | 0.16 (0.07–0.24) | 0.74 (0.32–1.15) |

**Abbreviations:** AUC – area under the receiver operating characteristic curve; NA – not applicable; NE – Not Estimable; SVM – support vector machine; TabPFN – Tabular Prior-Data Fitted Network.

**Supplementary Table S20.** Model performance in the sensitivity analysis restricted to patients with oral cavity and oropharyngeal tumours.

| **Approach** | **Model** | **Threshold** | **AUC (95% CI)** | **Sensitivity (95% CI)** | **Specificity (95% CI)** | **Accuracy (95% CI)** | **Precision (95% CI)** | **F1 score (95% CI)** | **Brier (95% CI)** | **Log loss (95% CI)** |
| --- | --- | --- | --- | --- | --- | --- | --- | --- | --- | --- |
| Class-weighted baseline | LR | 0.50 | 0.78 (0.61–0.95) | 0.68 (0.24–1.00) | 0.68 (0.52–0.83) | 0.68 (0.53–0.83) | 0.32 (0.05–0.60) | 0.41 (0.12–0.69) | 0.20 (0.12–0.27) | 0.57 (0.40–0.75) |
| Class-weighted baseline | SVM | 0.50 | 0.72 (0.46–0.98) | 0.00 (NE) | 1.00 (0.98–1.00) | 0.84 (0.78–0.89) | NA | 0.00 (NE) | 0.13 (0.10–0.17) | 0.43 (0.33–0.54) |
| Class-weighted baseline | RF | 0.50 | 0.58 (0.35–0.80) | 0.04 (0.00–0.25) | 0.88 (0.75–1.00) | 0.74 (0.62–0.86) | 0.02 (0.00–0.11) | 0.02 (0.00–0.12) | 0.20 (0.12–0.28) | 1.12 (0.00–2.50) |
| Class-weighted baseline | XGBoost | 0.50 | 0.61 (0.43–0.78) | 0.14 (0.00–0.42) | 0.82 (0.64–1.00) | 0.72 (0.57–0.87) | 0.19 (0.00–0.54) | 0.12 (0.00–0.35) | 0.24 (0.13–0.35) | 0.78 (0.41–1.16) |
| Class-weighted baseline | CatBoost | 0.50 | 0.69 (0.51–0.87) | 0.51 (0.06–0.96) | 0.64 (0.49–0.80) | 0.62 (0.49–0.76) | 0.23 (0.00–0.47) | 0.29 (0.04–0.54) | 0.22 (0.14–0.31) | 0.61 (0.38–0.83) |
| Baseline | TabPFN | 0.50 | 0.71 (0.51–0.91) | 0.00 (NE) | 0.98 (0.92–1.00) | 0.82 (0.75–0.90) | 0.00 (NE) | 0.00 (NE) | 0.14 (0.10–0.18) | 0.44 (0.33–0.56) |
| Threshold adjustment | LR | 0.49 | 0.78 (0.61–0.95) | 0.68 (0.24–1.00) | 0.68 (0.51–0.85) | 0.69 (0.54–0.83) | 0.32 (0.05–0.59) | 0.40 (0.12–0.69) | 0.20 (0.12–0.27) | 0.57 (0.40–0.75) |
| Threshold adjustment | SVM | 0.20 | 0.74 (0.50–0.98) | 0.70 (0.26–1.00) | 0.66 (0.51–0.81) | 0.67 (0.53–0.81) | 0.31 (0.06–0.55) | 0.40 (0.14–0.67) | 0.13 (0.10–0.16) | 0.42 (0.32–0.52) |
| Threshold adjustment | RF | 0.60 | 0.58 (0.35–0.80) | 0.04 (0.00–0.25) | 0.89 (0.76–1.00) | 0.75 (0.63–0.86) | 0.02 (0.00–0.12) | 0.02 (0.00–0.12) | 0.20 (0.12–0.28) | 1.12 (0.00–2.50) |
| Threshold adjustment | XGBoost | 0.77 | 0.61 (0.43–0.78) | 0.14 (0.00–0.42) | 0.87 (0.71–1.00) | 0.76 (0.63–0.88) | 0.22 (0.00–0.59) | 0.12 (0.00–0.36) | 0.24 (0.13–0.35) | 0.78 (0.41–1.16) |
| Threshold adjustment | CatBoost | 0.73 | 0.69 (0.51–0.87) | 0.08 (0.00–0.35) | 0.83 (0.66–1.00) | 0.71 (0.56–0.86) | 0.07 (0.00–0.24) | 0.05 (0.00–0.22) | 0.22 (0.14–0.31) | 0.61 (0.38–0.83) |
| Threshold adjustment | TabPFN | 0.29 | 0.71 (0.51–0.91) | 0.29 (0.00–0.71) | 0.80 (0.64–0.97) | 0.72 (0.58–0.87) | 0.22 (0.00–0.55) | 0.21 (0.00–0.53) | 0.14 (0.10–0.18) | 0.44 (0.33–0.56) |
| Data augmentation | LR | 0.50 | 0.79 (0.62–0.95) | 0.68 (0.24–1.00) | 0.69 (0.53–0.85) | 0.69 (0.54–0.85) | 0.32 (0.06–0.58) | 0.41 (0.13–0.70) | 0.19 (0.11–0.27) | 0.54 (0.33–0.74) |
| Data augmentation | SVM | 0.50 | 0.77 (0.58–0.95) | 0.68 (0.24–1.00) | 0.65 (0.49–0.81) | 0.66 (0.51–0.81) | 0.29 (0.07–0.51) | 0.39 (0.13–0.64) | 0.20 (0.12–0.27) | 0.55 (0.36–0.74) |
| Data augmentation | RF | 0.50 | 0.66 (0.42–0.90) | 0.55 (0.09–1.00) | 0.74 (0.57–0.90) | 0.71 (0.56–0.87) | 0.33 (0.00–0.65) | 0.38 (0.05–0.71) | 0.24 (0.12–0.36) | 2.51 (0.00–5.57) |
| Data augmentation | XGBoost | 0.50 | 0.65 (0.44–0.87) | 0.42 (0.00–0.87) | 0.72 (0.53–0.92) | 0.69 (0.52–0.85) | 0.26 (0.00–0.58) | 0.28 (0.00–0.60) | 0.25 (0.14–0.37) | 0.81 (0.43–1.19) |
| Data augmentation | CatBoost | 0.50 | 0.72 (0.55–0.90) | 0.60 (0.13–1.00) | 0.69 (0.52–0.86) | 0.69 (0.55–0.83) | 0.29 (0.02–0.56) | 0.36 (0.07–0.65) | 0.23 (0.13–0.33) | 0.64 (0.36–0.92) |
| Data augmentation | TabPFN | 0.50 | 0.76 (0.59–0.93) | 0.62 (0.15–1.00) | 0.69 (0.53–0.85) | 0.69 (0.54–0.83) | 0.30 (0.02–0.58) | 0.38 (0.08–0.67) | 0.21 (0.12–0.31) | 0.60 (0.35–0.85) |

**Abbreviations:** AUC – area under the receiver operating characteristic curve; CI – confidence interval; NA – not applicable; NE – Not Estimable; SVM – support vector machine; TabPFN – Tabular Prior-Data Fitted Network.

**Supplementary Table S21.** Model performance in the sensitivity analysis using mandibular V60Gy as the sole predictor.

| **Approach** | **Model** | **Threshold** | **AUC** | **Sensitivity** | **Specificity** | **Accuracy** | **Precision** | **F1 Score** | **Brier** | **Logloss** |
| --- | --- | --- | --- | --- | --- | --- | --- | --- | --- | --- |
| Class-weighted baseline | CatBoost | 0.50 | 0.80 (0.67–0.94) | 0.02 (0.00–0.12) | 0.97 (0.92–1.00) | 0.89 (0.85–0.93) | 0.01 (0.00–0.07) | 0.01 (0.00–0.09) | 0.08 (0.06–0.11) | 0.28 (0.20–0.36) |
| Class-weighted baseline | Logistic Regression | 0.50 | 0.84 (0.69–0.99) | 0.00 (NE) | 1.00 (0.99–1.00) | 0.92 (0.89–0.94) | NA | 0.00 (NE) | 0.07 (0.05–0.09) | 0.23 (0.17–0.30) |
| Class-weighted baseline | Random Forest | 0.50 | 0.63 (0.43–0.84) | 0.03 (0.00–0.16) | 0.93 (0.87–1.00) | 0.86 (0.80–0.92) | 0.02 (0.00–0.10) | 0.02 (0.00–0.12) | 0.10 (0.07–0.13) | 1.47 (0.30–2.64) |
| Class-weighted baseline | SVM | 0.50 | 0.46 (0.08–0.85) | 0.00 (NE) | 1.00 (NE) | 0.92 (0.89–0.94) | NA | 0.00 (NE) | 0.08 (0.06–0.10) | 0.29 (0.23–0.35) |
| Baseline | TabPFN | 0.50 | 0.82 (0.68–0.96) | 0.00 (NE) | 0.99 (0.96–1.00) | 0.91 (0.88–0.94) | NA | 0.00 (NE) | 0.07 (0.05–0.09) | 0.25 (0.19–0.32) |
| Class-weighted baseline | XGBoost | 0.50 | 0.72 (0.46–0.98) | 0.00 (NE) | 0.95 (0.89–1.00) | 0.87 (0.82–0.93) | NA | 0.00 (NE) | 0.09 (0.06–0.11) | 0.30 (0.20–0.40) |
| Threshold adjustment | CatBoost | 0.23 | 0.80 (0.67–0.94) | 0.18 (0.00–0.51) | 0.90 (0.82–0.98) | 0.84 (0.78–0.91) | 0.09 (0.00–0.25) | 0.11 (0.00–0.31) | 0.08 (0.06–0.11) | 0.28 (0.20–0.36) |
| Threshold adjustment | Logistic Regression | 0.14 | 0.84 (0.69–0.99) | 0.60 (0.17–1.00) | 0.81 (0.63–0.99) | 0.80 (0.65–0.95) | 0.26 (0.00–0.53) | 0.33 (0.06–0.59) | 0.07 (0.05–0.09) | 0.23 (0.17–0.30) |
| Threshold adjustment | Random Forest | 0.56 | 0.63 (0.43–0.84) | 0.03 (0.00–0.16) | 0.93 (0.87–1.00) | 0.86 (0.80–0.92) | 0.02 (0.00–0.10) | 0.02 (0.00–0.12) | 0.10 (0.07–0.13) | 1.47 (0.30–2.64) |
| Threshold adjustment | SVM | inf | 0.46 (0.08–0.85) | 0.68 (0.25–1.00) | 0.41 (0.00–0.85) | 0.44 (0.05–0.82) | 0.12 (0.00–0.28) | 0.18 (0.00–0.36) | 0.08 (0.06–0.10) | 0.30 (0.22–0.38) |
| Threshold adjustment | TabPFN | 0.15 | 0.82 (0.68–0.96) | 0.46 (0.04–0.88) | 0.86 (0.75–0.97) | 0.83 (0.73–0.93) | 0.25 (0.00–0.51) | 0.30 (0.03–0.56) | 0.07 (0.05–0.09) | 0.25 (0.19–0.32) |
| Threshold adjustment | XGBoost | 0.09 | 0.72 (0.46–0.98) | 0.49 (0.06–0.92) | 0.85 (0.74–0.96) | 0.82 (0.74–0.91) | 0.23 (0.00–0.48) | 0.29 (0.04–0.54) | 0.09 (0.06–0.11) | 0.30 (0.20–0.40) |
| Data augmentation | CatBoost | 0.50 | 0.78 (0.61–0.94) | 0.54 (0.08–1.00) | 0.75 (0.57–0.92) | 0.73 (0.58–0.88) | 0.16 (0.00–0.33) | 0.23 (0.01–0.45) | 0.19 (0.11–0.26) | 0.54 (0.33–0.76) |
| Data augmentation | Logistic Regression | 0.50 | 0.84 (0.69–0.99) | 0.74 (0.36–1.00) | 0.71 (0.58–0.85) | 0.72 (0.60–0.83) | 0.21 (0.04–0.38) | 0.30 (0.13–0.48) | 0.17 (0.11–0.23) | 0.51 (0.35–0.67) |
| Data augmentation | Random Forest | 0.50 | 0.73 (0.55–0.91) | 0.57 (0.12–1.00) | 0.75 (0.61–0.88) | 0.73 (0.61–0.86) | 0.19 (0.00–0.41) | 0.26 (0.02–0.51) | 0.22 (0.12–0.32) | 2.84 (0.64–5.05) |
| Data augmentation | SVM | 0.50 | 0.84 (0.69–0.99) | 0.74 (0.36–1.00) | 0.71 (0.57–0.85) | 0.72 (0.60–0.83) | 0.21 (0.04–0.38) | 0.30 (0.12–0.48) | 0.17 (0.11–0.23) | 0.51 (0.36–0.67) |
| Data augmentation | TabPFN | 0.50 | 0.80 (0.64–0.96) | 0.65 (0.22–1.00) | 0.67 (0.50–0.84) | 0.67 (0.53–0.81) | 0.15 (0.00–0.31) | 0.23 (0.07–0.39) | 0.19 (0.12–0.26) | 0.52 (0.35–0.70) |
| Data augmentation | XGBoost | 0.50 | 0.76 (0.57–0.96) | 0.61 (0.15–1.00) | 0.74 (0.60–0.89) | 0.73 (0.62–0.85) | 0.16 (0.02–0.30) | 0.24 (0.05–0.44) | 0.21 (0.12–0.30) | 0.67 (0.38–0.96) |

**Abbreviations:** AUC – area under the receiver operating characteristic curve; Gy – gray; NA – not applicable; NE – Not Estimable; SVM – support vector machine; TabPFN – Tabular Prior-Data Fitted Network; V60Gy – percentage of the mandibular volume receiving at least 60 Gy;. “inf” indicates that the mean optimized threshold was infinite in some resampling iterations.

**Supplementary Table S22.** Model performance in the sensitivity analysis using oral cavity tumour location as the sole predictor.

| **Approach** | **Model** | **Threshold** | **AUC** | **Sensitivity** | **Specificity** | **Accuracy** | **Precision** | **F1 Score** | **Brier** | **Logloss** |
| --- | --- | --- | --- | --- | --- | --- | --- | --- | --- | --- |
| **Class-weighted baseline** | CatBoost | 0.50 | 0.81 (0.64–0.97) | 0.78 (0.46–1.00) | 0.83 (0.75–0.91) | 0.82 (0.75–0.90) | 0.32 (0.11–0.53) | 0.43 (0.22–0.63) | 0.16 (0.12–0.21) | 0.52 (0.41–0.64) |
| **Class-weighted baseline** | Logistic Regression | 0.50 | 0.81 (0.64–0.97) | 0.78 (0.46–1.00) | 0.83 (0.75–0.91) | 0.82 (0.75–0.90) | 0.32 (0.11–0.53) | 0.43 (0.22–0.63) | 0.16 (0.12–0.21) | 0.52 (0.41–0.63) |
| **Class-weighted baseline** | Random Forest | 0.50 | 0.81 (0.64–0.97) | 0.78 (0.46–1.00) | 0.83 (0.75–0.91) | 0.82 (0.75–0.90) | 0.32 (0.11–0.53) | 0.43 (0.22–0.63) | 0.15 (0.10–0.19) | 0.54 (0.03–1.04) |
| **Class-weighted baseline** | SVM | 0.50 | 0.81 (0.64–0.97) | 0.00 (NE) | 1.00 (NE) | 0.92 (0.89–0.94) | NA | 0.00 (NE) | 0.07 (0.04–0.09) | 0.24 (0.15–0.32) |
| **Baseline** | TabPFN | 0.50 | 0.80 (0.63–0.97) | 0.00 (NE) | 1.00 (NE) | 0.92 (0.89–0.94) | NA | 0.00 (NE) | 0.07 (0.04–0.09) | 0.24 (0.14–0.35) |
| **Class-weighted baseline** | XGBoost | 0.50 | 0.81 (0.64–0.97) | 0.78 (0.46–1.00) | 0.83 (0.75–0.91) | 0.82 (0.75–0.90) | 0.32 (0.11–0.53) | 0.43 (0.22–0.63) | 0.16 (0.12–0.21) | 0.52 (0.41–0.64) |
| **Threshold adjustment** | CatBoost | 0.81 | 0.81 (0.64–0.97) | 0.78 (0.46–1.00) | 0.83 (0.75–0.91) | 0.82 (0.75–0.90) | 0.32 (0.11–0.53) | 0.43 (0.22–0.63) | 0.16 (0.12–0.21) | 0.52 (0.41–0.64) |
| **Threshold adjustment** | Logistic Regression | 0.80 | 0.81 (0.64–0.97) | 0.78 (0.46–1.00) | 0.83 (0.75–0.91) | 0.82 (0.75–0.90) | 0.32 (0.11–0.53) | 0.43 (0.22–0.63) | 0.16 (0.12–0.21) | 0.52 (0.41–0.63) |
| **Threshold adjustment** | Random Forest | 0.78 | 0.81 (0.64–0.97) | 0.78 (0.46–1.00) | 0.83 (0.75–0.91) | 0.82 (0.75–0.90) | 0.32 (0.11–0.53) | 0.43 (0.22–0.63) | 0.15 (0.10–0.19) | 0.54 (0.03–1.04) |
| **Threshold adjustment** | SVM | inf | 0.77 (0.55–0.99) | 0.74 (0.39–1.00) | 0.84 (0.75–0.92) | 0.82 (0.75–0.90) | 0.31 (0.10–0.52) | 0.40 (0.18–0.62) | 0.07 (0.04–0.09) | 0.25 (0.15–0.34) |
| **Threshold adjustment** | TabPFN | 0.28 | 0.80 (0.63–0.97) | 0.51 (0.03–0.99) | 0.87 (0.70–1.00) | 0.84 (0.70–0.98) | 0.29 (0.06–0.52) | 0.27 (0.00–0.54) | 0.07 (0.04–0.09) | 0.24 (0.14–0.35) |
| **Threshold adjustment** | XGBoost | 0.82 | 0.81 (0.64–0.97) | 0.78 (0.46–1.00) | 0.83 (0.75–0.91) | 0.82 (0.75–0.90) | 0.32 (0.11–0.53) | 0.43 (0.22–0.63) | 0.16 (0.12–0.21) | 0.52 (0.41–0.64) |
| **Random oversampling** | CatBoost | 0.50 | 0.81 (0.64–0.97) | 0.78 (0.46–1.00) | 0.83 (0.75–0.91) | 0.82 (0.75–0.90) | 0.32 (0.11–0.53) | 0.43 (0.22–0.63) | 0.17 (0.12–0.21) | 0.53 (0.42–0.65) |
| **Random oversampling** | Logistic Regression | 0.50 | 0.81 (0.64–0.97) | 0.78 (0.46–1.00) | 0.83 (0.75–0.91) | 0.82 (0.75–0.90) | 0.32 (0.11–0.53) | 0.43 (0.22–0.63) | 0.17 (0.12–0.21) | 0.53 (0.43–0.63) |
| **Random oversampling** | Random Forest | 0.50 | 0.81 (0.64–0.97) | 0.78 (0.46–1.00) | 0.83 (0.75–0.91) | 0.82 (0.75–0.90) | 0.32 (0.11–0.53) | 0.43 (0.22–0.63) | 0.17 (0.12–0.21) | 0.60 (0.10–1.09) |
| **Random oversampling** | SVM | 0.50 | 0.81 (0.64–0.97) | 0.78 (0.46–1.00) | 0.83 (0.75–0.91) | 0.82 (0.75–0.90) | 0.32 (0.11–0.53) | 0.43 (0.22–0.63) | 0.17 (0.12–0.21) | 0.53 (0.43–0.63) |
| **Random oversampling** | TabPFN | 0.50 | 0.81 (0.64–0.98) | 0.78 (0.46–1.00) | 0.83 (0.75–0.91) | 0.82 (0.75–0.90) | 0.32 (0.11–0.53) | 0.43 (0.22–0.63) | 0.16 (0.11–0.21) | 0.51 (0.39–0.63) |
| **Random oversampling** | XGBoost | 0.50 | 0.81 (0.64–0.97) | 0.78 (0.46–1.00) | 0.83 (0.75–0.91) | 0.82 (0.75–0.90) | 0.32 (0.11–0.53) | 0.43 (0.22–0.63) | 0.17 (0.12–0.21) | 0.53 (0.42–0.64) |

**Abbreviations:** AUC – area under the receiver operating characteristic curve; NA – not applicable; NE – Not Estimable; SVM – support vector machine. “inf” indicates that the mean optimized threshold was infinite in some resampling iterations.

**Supplementary Table S23.** Performance of the naive oral-cavity rule for predicting osteoradionecrosis.

| **Prediction rule** | **TP** | **FP** | **TN** | **FN** | **Sensitivity** | **Specificity** | **Accuracy** | **Precision** | **F1 score** | **ROC-AUC** |
| --- | --- | --- | --- | --- | --- | --- | --- | --- | --- | --- |
| Predict ORN when the primary tumour is located in the oral cavity | 6 | 15 | 74 | 2 | 0.750 | 0.831 | 0.825 | 0.286 | 0.414 | 0.791 |

The rule was evaluated directly in the complete cohort without model training or cross-validation. **Abbreviations:** FN – false negatives; FP – false positives; ORN – osteoradionecrosis; ROC-AUC – area under the receiver operating characteristic curve; TN – true negatives; TP – true positives.
